# Antigen-specific oncolytic MV-based tumor vaccines through presentation of selected tumor-associated antigens on infected cells or virus-like particles

**DOI:** 10.1038/s41598-017-16928-8

**Published:** 2017-12-04

**Authors:** Stefan Hutzler, Stephanie Erbar, Robert A. Jabulowsky, Jan R. H. Hanauer, Jürgen H. Schnotz, Tim Beissert, Bianca S. Bodmer, Regina Eberle, Klaus Boller, Thorsten Klamp, Ugur Sahin, Michael D. Mühlebach

**Affiliations:** 1 Section Product Testing of IVMPs, Division of Veterinary Medicine, Paul-Ehrlich-Institut, Paul-Ehrlich-Str. 51-59, D-63225 Langen, Germany; 2 Biopharmaceutical New Technologies (BioNTech) Corporation, An der Goldgrube 12, D-55131 Mainz, Germany; 3 TRON – Translational Oncology at the University Medical Center of the Johannes Gutenberg University gGmbH, Freiligrathstr. 12, D-55131 Mainz, Germany; 4 Section Morphology, Division of Immunology, Paul-Ehrlich-Institut, Paul-Ehrlich-Str. 51-59, D-63225 Langen, Germany

## Abstract

Recombinant vaccine strain-derived measles virus (MV) is clinically tested both as vaccine platform to protect against other pathogens and as oncolytic virus for tumor treatment. To investigate the potential synergism in anti-tumoral efficacy of oncolytic and vaccine properties, we chose Ovalbumin and an ideal tumor antigen, claudin-6, for pre-clinical proof of concept. To enhance immunogenicity, both antigens were presented by retroviral virus-like particle produced *in situ* during MV-infection. All recombinant MV revealed normal growths, genetic stability, and proper expression and presentation of both antigens. Potent antigen-specific humoral and cellular immunity were found in immunized MV-susceptible IFNAR^−/−^-CD46Ge mice. These immune responses significantly inhibited metastasis formation or increased therapeutic efficacy compared to control MV in respective novel *in vivo* tumor models using syngeneic B16-hCD46/mCLDN6 murine melanoma cells. These data indicate the potential of MV to trigger selected tumor antigen-specific immune responses on top of direct tumor lysis for enhanced efficacy.

## Introduction

Despite significant advances in tumor therapy, the efficacy of classic therapeutic options like surgery, radio-, chemo-, or antibody-therapy for patients with advanced stage solid tumor entities remains limited. In the past few years, novel treatment options have been developed that prime the immune system for tumor cell eradication. Amongst these, the presentation of tumor-associated antigens (TAA) has been shown to be a promising approach to induce potent and persisting antigen-specific T-cell responses. For this purpose, immunotherapies aim to break tolerance of the immune system against endogenous self- or to even target patient-specific mutant neo-antigens^1^. Cancer vaccines already licensed or currently in clinical trials comprise several different antigenic formats including vaccines targeting HPV antigens as the causative agent of cervical carcinoma^2^, dendritic cells loaded with antigens/peptides^3^, antigen-adjuvant conjugates (e.g. Sipuleucel-T/Provenge™)^4^, or antigen-encoding modified RNAs^5^.

Also viruses are considered as tumor-lytic cancer therapeutics. Application of unmodified wild-type viruses has only rarely been successful, but was sometimes accompanied by significant disease caused by the infection. The advent of recombinant DNA technologies allowed rational development of viruses tailored for the specific lysis of tumor cells, so called oncolytic viruses (OVs). OVs have been derived from at least nine different virus families and have broadly entered early to advanced phase clinical trials^6^. While OVs have originally been developed for direct tumor cell lysis due to their inherent cytotoxicity, the potential contribution of the immune system to treatment efficacy seemed ambiguous. On the one hand, anti-viral immunity can inhibit oncolysis. On the other hand, at least some OVs trigger anti-tumoral immune responses, which has led to a change of the “oncolytic dogma”^7^. Local inflammation due to the release of pathogen- and danger-associated molecular pattern (PAMPs and DAMPs, respectively) during oncolysis transforms the originally immune-suppressive microenvironment into an immune-stimulatory one, triggering not only anti-viral, but also anti-tumoral immune responses, which may also act on distant tumor sites.

One prominent example for cancer immunotherapy by oncolytic OV is Talimogene laherparepvec (T-VEC, Imlygic™)^8^, a genetically modified herpes simplex virus expressing GM-CSF recently licensed for the treatment of melanoma^9^. Also GM-CSF encoding recombinant vaccine strain-derived measles viruses (MV) have been pre-clinically tested^10,11^, but only marker-gene encoding MVs are currently developed in several phase I and two phase II clinical trials (www.clinicaltrials.gov). Nevertheless, the co-expression of immune-stimulants significantly enhanced the efficacy in mouse tumor models^10,12^. Although indicating the potential benefit of immune-stimulation, the (natural) selection of the tumor antigen(s), which the induced immunity is directed against, is presently not understood. This implies the risk of induction of autoimmune pathogenicity by selection of an antigen being also expressed on healthy tissue, especially, if combined with immuno-oncologic agents such as check-point inhibitors. To better focus induced anti-tumoral responses, other OVs, i.e. vesicular stomatitis virus (VSV) or Maraba virus, have been equipped with expression cassettes encoding selected TAAs to direct immunity to those. However, these desired responses could only be induced by employing a heterologous prime-boost scheme using an adenoviral vector as prime, yet, since otherwise anti-OV immunity seemed too dominant^13,14^. In parallel, recombinant MV is developed as a vaccine platform by encoding foreign antigens in extra expression cassettes, so called additional transcription units (ATUs). MV expressing antigens of a range of different pathogens have been generated and have shown protection in pre-clinical models^15,16^ as well as safety and immunogenicity in a phase I clinical trial^17^.

Here, we aimed to combine the oncolytic and the excellent vaccine platform properties of vaccine strain-derived MV with an ideal TAA to induce potent anti-tumoral immune responses, but no immunopathogenicity. The oncofetal tight junction molecule Claudin 6 (CLND6) constitutes such a TAA for anti-cancer immunotherapy. Physiological expression of CLDN6 is solely restricted to early stages during embryogenesis or in the placenta^18^. Being virtually absent from any normal tissue due to transcriptional silencing in adult healthy tissues^19–24^ CLDN6 is aberrantly and frequently expressed in various types of cancers of high medical need such as ovarian, lung, gastric breast, prostate, and pediatric cancers^21,22,24,25^. Safety of CLDN6 as a promising anti-cancer immunotherapy target is suggested by a clinical phase I/II trial using an anti-CLDN6 monoclonal antibody in advanced ovarian carcinoma patients (NCT02054351)^26^.

Recombinant MV was chosen as oncolytic platform of choice, since MV is one of the platforms well progressing in clinical trials, of note also against ovarian carcinoma – a target entity for CLDN6 vaccination as outlined above. In these studies, MV has shown an excellent safety profile during application of up to 1 × 10^11^ infectious particles to terminal cancer patients. Thereby, first evidence of clinical efficacy has been generated^27^. Moreover, the MV backbone used here is identical to the measles vaccine strain, which is extremely safe without causing latency^28^, very immunogenic thereby inducing long-lasting immunity^29,30^, and, in contrast to e.g. poxviral vectors, boostable^31,32^. The potential application as a vaccine platform^33^ with frontrunner targets chikungunya virus and Zika virus is also progressing through clinical development^17^ and has entered phase II, too (NCT02861586). This specific profile of dual applicability as oncolytic virus as well as a vaccine platform thus mandated further investigation of potential synergism of both properties in one candidate.

To further increase chances to induce anti-tumoral immunity against this TAA without the need of a heterologous prime-boost, we choose to enhance the TAAs immunogenicity by optimized presentation. One format of highly immunogenic antigen-presentation are virus-like particles (VLPs). VLPs are derived from viruses, but are not infectious. VLPs can incorporate and present selected antigens of choice on their surface. For example, hepatitis B virus core antigen (HBcAg)-based VLPs presenting an immunogenic peptide of the TAA Claudin 18 isoform 2 (CLDN18.2)^34^ or murine leukemia virus (MLV)-derived VLPs presenting immunogenic peptides of the amyloid beta protein^35^ are able to effectively break immune tolerance against these auto-antigens. Therefore, we chose to test TAA-presentation on gag-VLPs as an additional immunogenic stimulus, since no other oncolytic virus has shown induction of antigen-specific anti-tumoral immunity without a heterologous prime, yet, if just encoding a TAA^36^.

**Figure 1:**
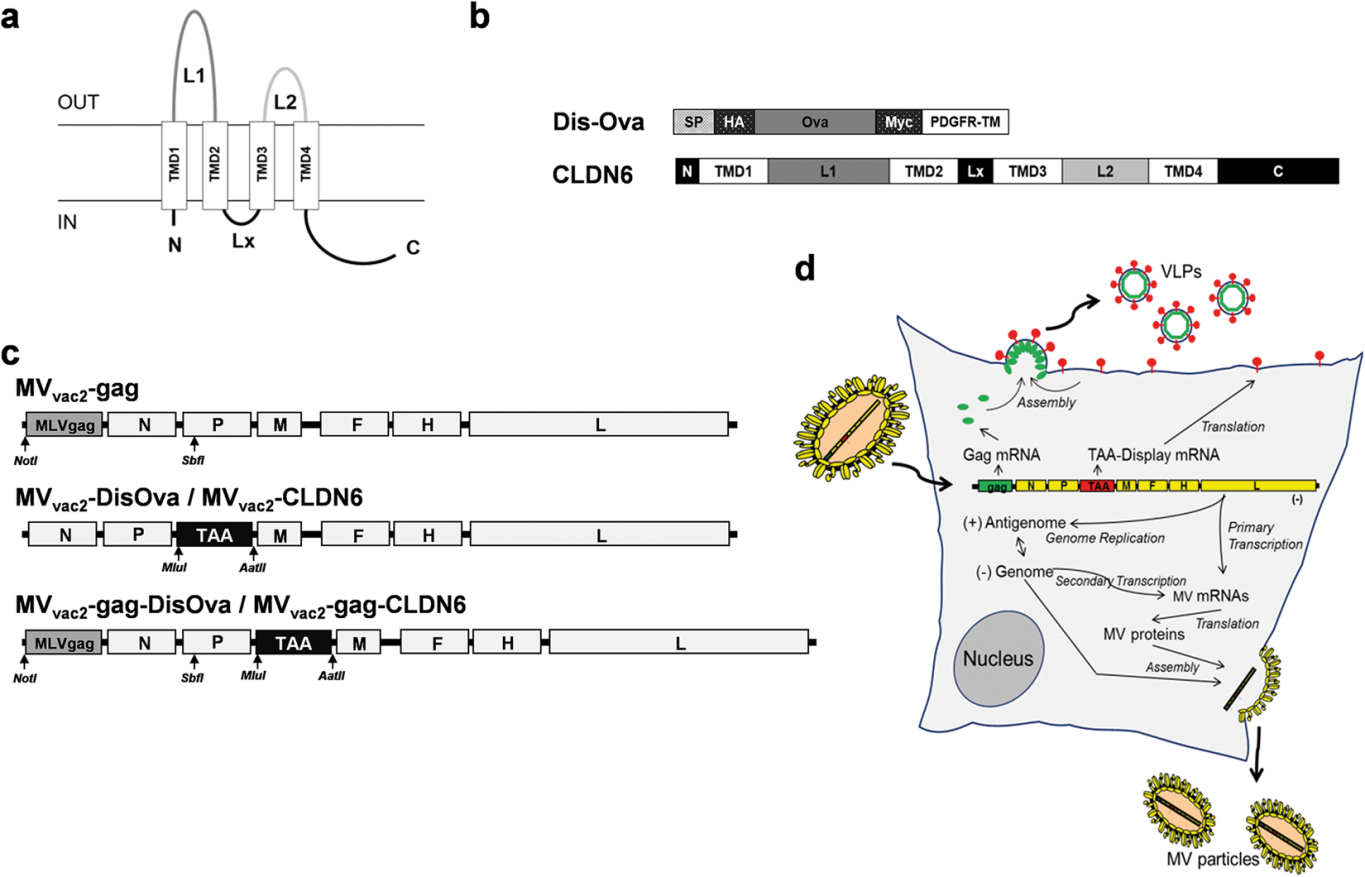
Generation of MV presenting native or particle-associated tumor-associated antigens (TAAs). (**a**) Schematic depiction and membrane organization of CLDN6, a typical four-transmembrane protein. (**b**) CLDN6 and a membrane-bound, extracellular version of Ovalbumin (DisOva) were generated for MV-associated presentation of tumor-associated antigens (TAAs). CLDN6 or Ovalbumin encoding genes are depicted in light grey, tags in dark grey and transmembrane domains (TMD) in white. SP, signal-peptide; N, N-terminus; C, C-terminus; L, intracellular loop. (**c**) Schematic depiction of recombinant MV_vac2_-derived genomes with annotated viral genes in light grey. An additional gene cassette was inserted in pre-N gene position for the expression of MLV-derived VLPs (top). The TAA-encoding versions were expressed in post-P gene position either without (middle) or with (bottom) an additional gene cassette in pre-N gene position for the expression of MLV-derived VLPs (MLVgag). TAA- (black), MLVgag-encoding genes (grey) and MV viral gene cassettes (white) are annotated. Restriction sites used for cloning of additional genes are indicated in italics. (**d**) Schematic depiction of infection, Ag-presentation, and life-cycle of TAA-encoding MV giving rise to VLP-presentation. Yellow, MV genes and gene products; green, MLV-gag gene and Gag protein; red, TAA-display gene or gene product.

In this study, recombinant MVs were generated that express CLDN6 alone or in combination with MLV Gag, sufficient to generate retroviral VLPs in Gag-expressing cells (Fig. 1d). For initial proof of concept, chicken Ovalbumin (Ova) was chosen to facilitate assessment of immune reactions triggered by MV presenting antigens on membranes of infected cells or by MLV-VLPs. Recombinant MVs encoding a membrane-bound version of Ova (DisOva) or murine CLDN6 with or without MLV-Gag were generated and characterized *in vitro*: All recombinant viruses expressed the respective antigens either cell- or VLP-associated, and replicated with unmodified vaccine’s efficacy. In MV-susceptible mice, these modified MVs triggered significant cellular and humoral immune responses against either Ova or autologous CLDN6. This anti-tumoral immunity inhibited metastasis formation and was also effective against pre-established solid tumors in a B16-mCLDN6/hCD46 melanoma model demonstrating therapeutic efficacy of this new approach in advanced *in vivo* tumor models that allowed for the first time direct interaction of the oncolytic MV with immune cells, as to be expected also in the human system.

## Results

### Generation and characterization of recombinant MV_vac2_ encoding different forms of the Ova-antigen

To test oncolytic MV as flexible immunotherapeutic platform to present tumor associated antigens alone or in a particulate manner on virus-like particles (VLPs), we used Ovalbumin (Ova) as model antigen. Ova was chosen since especially the cellular immune-responses and respective immune-dominant peptides in mice such as used in our studies are well known and can thus considerably support characterization of such responses. For this purpose, Ova was genetically fused to the transmembrane domain of the platelet-derived growth factor receptor (PDGFR-TM) giving rise to a membrane-bound extracellular variant (DisOva) that should be readily incorporated into retroviral particles (Fig. 1b). A recombinant MV (MV_vac2_-gag-DisOva) encoding DisOva in an additional transcription unit (ATU) following the P gene in combination with a second, MLV Gag*-*encoding transgene cassette in front of the viral N gene, which give rise to *in situ* produced VLPs, was also generated (Fig. 1c,d). To analyze the immunogenicity of particle-associated antigen presentation by the resulting Ova-VLPs in comparison to exclusive surface antigen expression, a variant only carrying DisOva (MV_vac2_-DisOva) as well as a recombinant MV just encoding MLV-Gag (MV_vac2_-gag) for the release of bare VLPs have been generated (Fig. 1c). All recombinant viruses were successfully rescued and amplified up to passage 10 (P10) in Vero cells with comparable titers of up to 5 × 10^7^ TCID_50_/ml.

**Figure 2:**
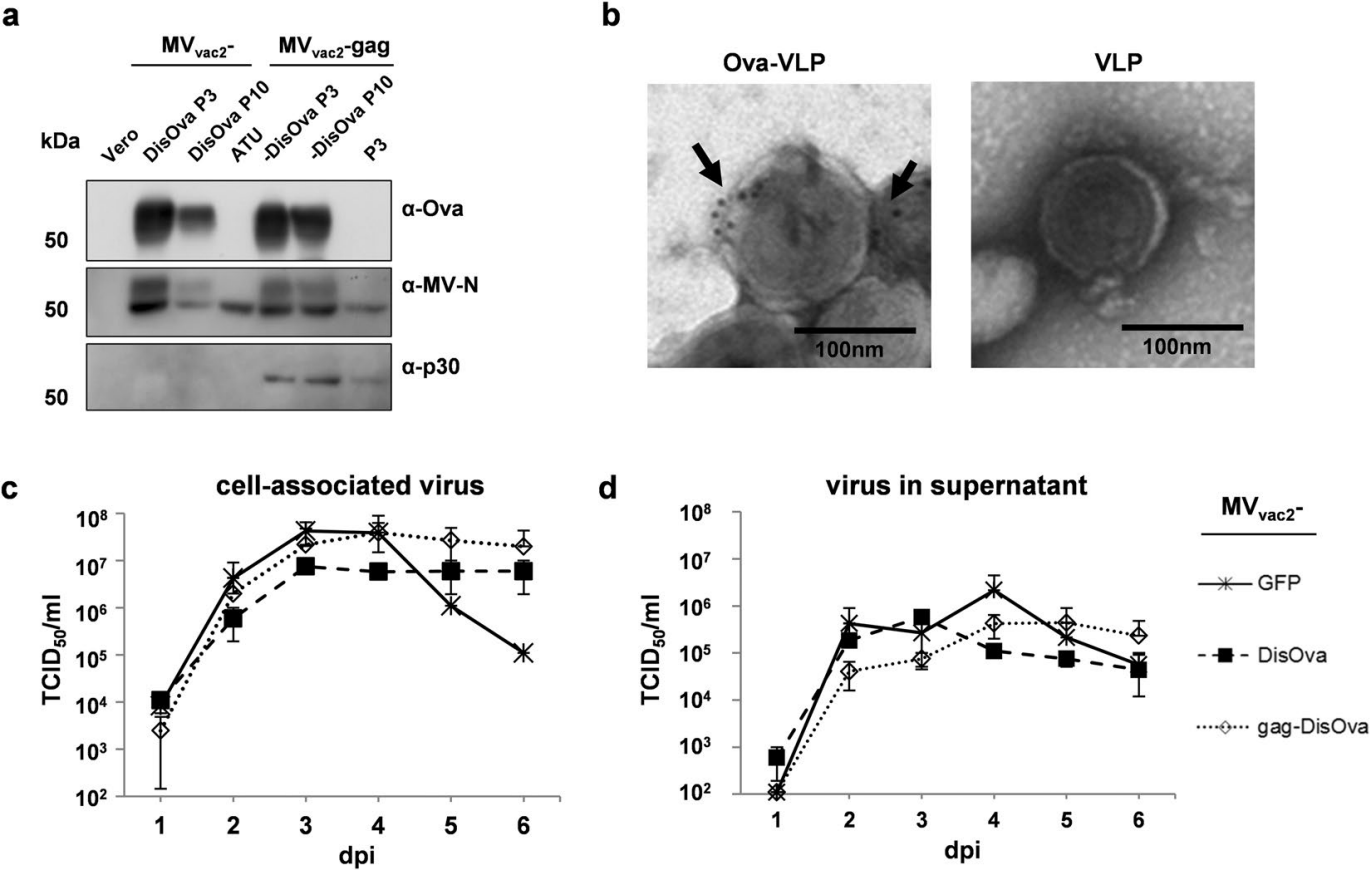
Characterization of Ovalbumin-presenting recombinant MV_vac2_. (**a**) Immunoblot analysis of Vero cells infected at an MOI of 0.03 with MV_vac2_ or MV_vac2_-gag expressing membrane-bound extracellular version of Ovalbumin (DisOva) compared to vaccine expressing no antigen (MV_vac2_-ATU) in virus passages 3 (P3) or 10 (P10). Uninfected Vero cells served as control. Blots were probed as indicated. (**b**) Immunoelectron microscopic analysis of MLV-derived VLPs purified from supernatants of virus-infected Vero cells. VLPs displaying Ovalbumin or pure VLPs, as depicted above images, analyzed after fixation and probing for Ova. Arrows depict Ova-specific labelling. Scale bar: 100 nm. (**c**,**d**) Growth kinetics of recombinant MV (P3) on Vero cells infected at an MOI of 0.03 with indicated viruses. Cell-associated virus titers (**c**) as well as virus titers in the supernatant (**d**) of samples prepared at indicated time points post infection were titrated on Vero cells. Means and standard deviations of three independent experiments. dpi, days post infection; TCID_50_, tissue culture infectious dose 50.

The stability of the *ova* and *gag* gene cassettes additionally introduced into the viral genomes was confirmed by sequencing after P3 and P10 (data not shown). Expression of DisOva was also verified for viruses in P3 or P10. Antigen-expression was clearly detectable in Vero cells infected with either MV_vac2_-DisOva or MV_vac2_-gag-DisOva, and stable over several passages (Fig. 2a). Moreover, electron microscopy combined with immunogold-labeling against Ova revealed incorporation of DisOva into retroviral VLPs isolated from the supernatant of MV_vac2_-gag-DisOva infected cells, while no Ova was detected on VLPs isolated from the supernatant of MV_vac2_-gag infected cells, as expected (Fig. 2b).

To assess the replication efficiency of the different vectors, multi-step growth kinetics of cell-associated and released virus were performed following infection at low MOI = 0.03 (Fig. 2c,d). Compared to the GFP-encoding control virus MV_vac2_-GFP, MVs encompassing the DisOva transgene cassette together with or without MLV *gag* grew with similar kinetics and reached similar maximum titers as the control virus. Thus, cloning and rescue of both MVs-derived vectors resulted in expression of the inserted antigen(s) in infected cells without impact on viral replication or genetic stability.

### Antigen-specific cellular immunity against vector and transgene is induced by Ova-presenting MV

**Figure 3:**
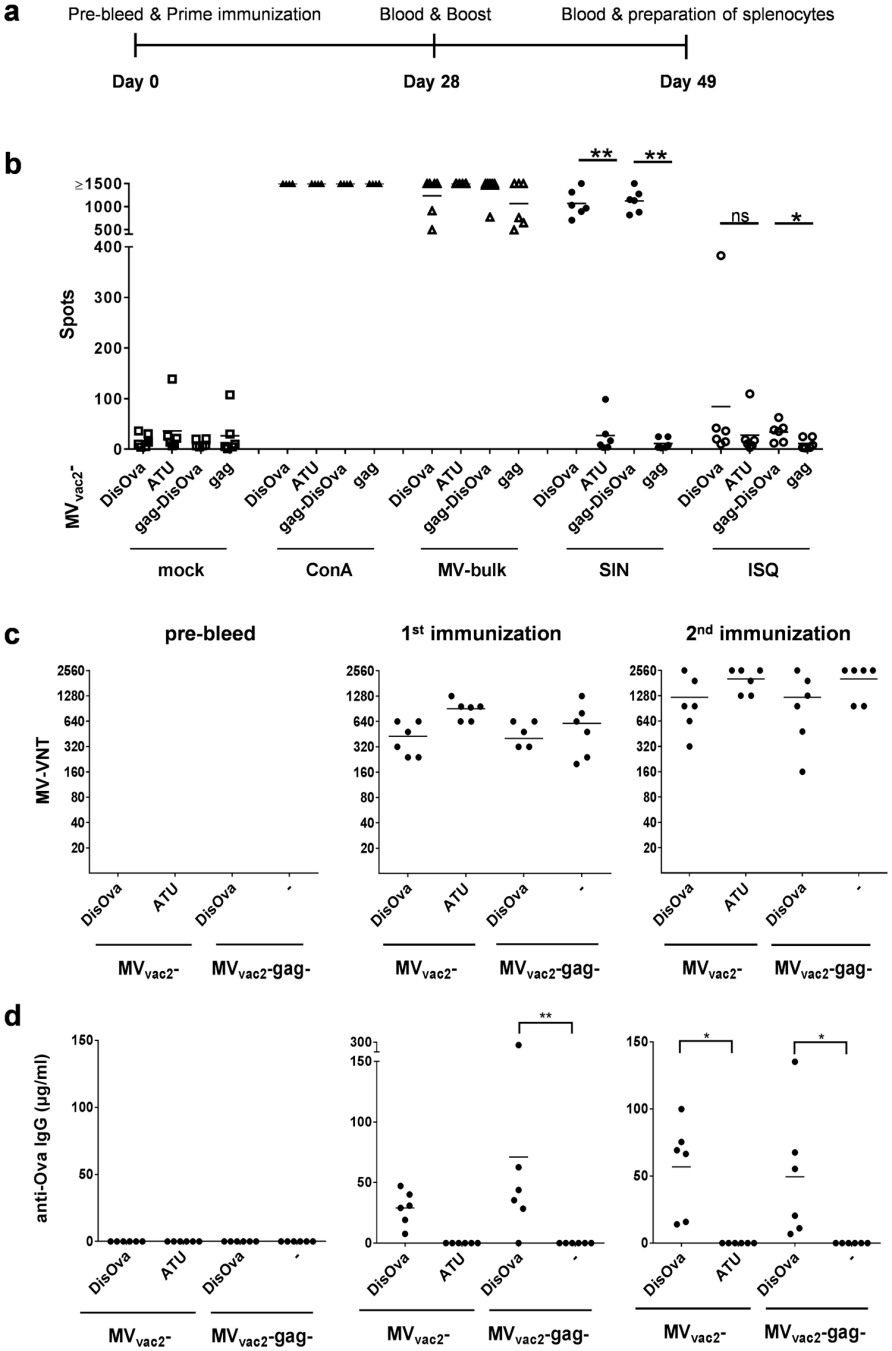
Antigen-specific cellular and humoral immunity induced by Ovalbumin-presenting MV. (**a**) Schematic depiction of immunization experiments (**b**) IFN-γ ELISpot analysis using splenocytes of vaccinated mice with indicated viruses expressing membrane-bound, extracellular Ovalbumin (MV_vac2_-DisOva, MV_vac2_-gag-DisOva). Vaccines expressing no antigen (MV_vac2_-ATU) or just naked VLPs (MV_vac2_-gag) served as negative controls. Splenocytes isolated 21 days after boost immunization were analyzed for IFN-γ-production after incubation with ConA, viral antigen (MV bulk), peptides (SIN, ISQ), or without stimulation (mock). (**c**) Virus neutralizing titers (VNT) in vaccinated animals’ sera were analyzed for neutralization of MV. Lower limit of detection (LLOD) = VNT 20. (**d**) Ovalbumin-specific antibody concentration in serum samples of vaccinated mice was determined using indirect ELISA. Dots represent single animals (n = 5–6); horizontal line represents mean per group. Statistical differences between groups were assessed by Mann-Whitney test (**b**) or two-way ANOVA (**d**); ns, not significant; *P < 0.05; **P < 0.01.

To analyze the induction of vector- or Ova-specific cellular immune responses, splenocytes of IFNAR^−/−^-CD46Ge mice vaccinated with MV_vac2_-DisOva or MV_vac2_-gag-DisOva (controls: MV_vac2_-ATU empty vaccine or MV_vac2_-gag presenting naked retroviral particles) in a prime-boost protocol (Fig. 3a) were isolated and analyzed for vector- and antigen-specific IFN-γ secretion by ELISpot assay. Isolated splenocytes were stimulated by MV bulk antigen to detect vector-specific T-cell responses, the MHC-I restricted SIINFEKL (SIN) Ova-peptide to detect specific CD8^+^ T-cell responses, the MHC-II restricted ISQAVHAAHAEINEAGR (ISQ) Ova-peptide to detect specific CD4^+^ T-cell responses, or concanavalin A (ConA) as control for general T-cell reactivity.

Splenocytes of all mice revealed a similar basic reactivity to broad T-cell stimulation by ConA, as shown by the high numbers of IFN-γ secreting cells with more than 750 IFN-γ secreting cells per 5 × 10^5^ splenocytes (Fig. 3b). Assessment of anti-vector cellular immunity using MV bulk antigen as stimulus revealed robust induction of IFN-γ secreting cells (>250/5 × 10^5^) irrespective of additionally encoded antigens or particles. Thus, there was no impact on general or vector-specific T-cell reactivity associated with the different vaccine candidates.

ELISpot assays using splenocytes of animals vaccinated with membrane-bound DisOva-encoding MVs with or without MLV-Gag revealed approximately 600 IFN-γ secreting cells per 5 × 10^5^ splenocytes after specific re-stimulation with SIINFEKL peptide (SIN). In contrast, control mice vaccinated with MV_vac2_-ATU or MV_vac2_-gag revealed only background responses of around 10 IFN-γ producing cells per 5 × 10^5^ splenocytes, comparable to non-stimulated splenocytes (mock). Of note, Ova-specific re-stimulation with ISQ triggered statistically significant results with around 15–45 IFN-γ secreting cells per 5 × 10^5^ splenocytes only for splenocytes of mice immunized with MV presenting DisOva on the surface of *in situ* produced particles (Fig. 3b). These data argue for robust induction of antigen-specific cytotoxic T-cell responses by MV encoding an immunogenic antigen, in general, whereas CD4^+^ T helper cells, which can be re-stimulated by the ISQ peptide, were significantly induced only by particle-associated antigen, as expected.

### Antigen-specific humoral immunity against vector and transgene is induced by Ova-presenting MV

To elucidate the extent of humoral immune responses, mice immunized as described were used to determine antibody prevalence in serum. First, induction of neutralizing antibodies against MV was analyzed (Fig. 3c). All groups immunized with recombinant MV developed mean virus neutralizing titers (VNT) of 500–700 already after the first immunization (Fig. 3c, middle panel). These mean titers increased after the second immunization to 1,200–2,560 (Fig. 3c, right panel). Again, no differences between the individual candidate vaccines became evident.

To assess the induction of Ova-specific humoral immune responses, antibodies binding to Ova were quantified by indirect ELISA (Fig. 3d). Ova-specific antibodies were found already after the first immunization (Fig. 3d, middle panel), with significantly increased titers over the control group only for vaccines encoding particle-associated antigen. Both variants of DisOva-encoding MV reached mean antibody concentrations of more than 50 µg/ml at day 21 after the booster vaccination. In summary, the MV-derived model vaccines expressing Ova either on the infected cells alone or in combination with presentation on *in situ* generated retroviral VLPs induced strong Ova-specific cellular and humoral immune responses.

### Generation and characterization of recombinant MV_vac2_ differently presenting murine CLDN6

**Figure 4:**
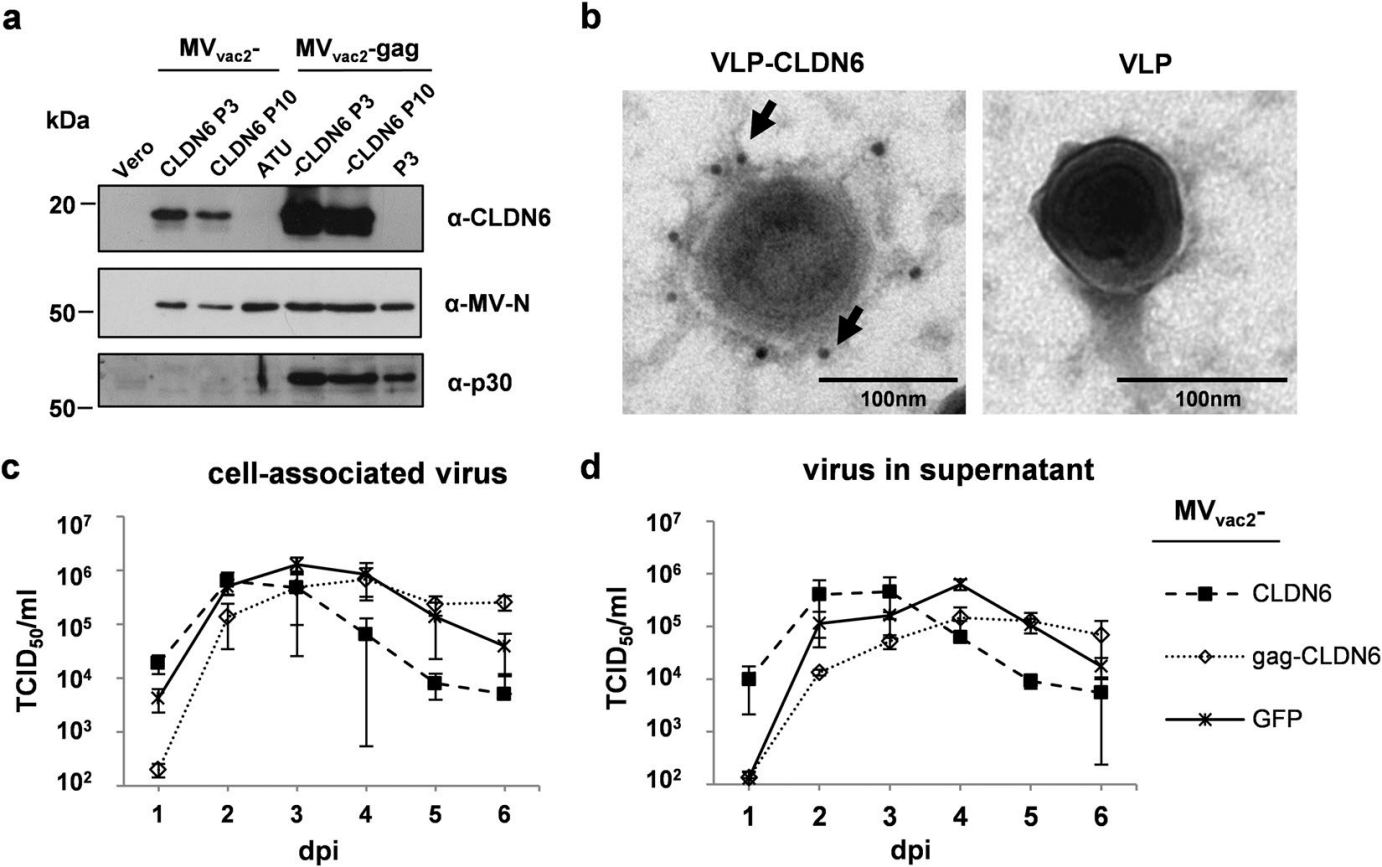
Characterization of CLDN6-presenting recombinant MV_vac2_. (**a**) Immunoblot analysis of Vero cells infected at an MOI of 0.03 with MV_vac2_ or MV_vac2_-MLVgag expressing CLDN6 in virus passages 3 (P3) or 10 (P10). Uninfected Vero cells served as control. Blots were probed as indicated. (**b**) Immunoelectron microscopic analysis of MLV-derived VLPs purified from supernatant (SN) of virus-infected Vero cells. VLPs displaying CLDN6 or only VLPs, as depicted above images, analyzed after fixation and probing for CLDN6 using a specific mAb. Arrows depict CLDN6-specific labelling. Scale bar: 100 nm. (**c**,**d**) Growth kinetics of recombinant MV (P3) on Vero cells infected at an MOI of 0.03 with indicated viruses. Cell-associated (**c**) as well as virus titers in the supernatant (**d**) of samples prepared at indicated time points post infection were titrated on Vero cells. Means and standard deviations of three independent experiments. dpi, days post infection.

After demonstrating the efficient induction of potent immune responses against the model-antigen Ova, we next assessed the immunogenicity of a prototypic MV-derived tumor vaccine encoding an autologous TAA. As described, the TAA Claudin-6 (CLDN6, Fig. 1a), a typical four-transmembrane protein, was chosen for pre-clinical proof of concept, as CLDN6 fulfills all criteria for an ideal tumor vaccination antigen. The murine gene homologue was used to fully mimic the auto-antigen situation. The full-length gene of mCLDN6 (Fig. 1b) was inserted into the ATU following the P gene cassette of the respective vector plasmids, yielding MV_vac2_-CLDN6 and MV_vac2_-gag-CLDN6 (Fig. 1c) after virus rescue. Individual virus clones were generated and characterized *in vitro* as described for DisOva-encoding viruses. The viruses were easily amplified up to P10 with titers of up to 4×10^7^ TCID_50_/ml, revealing stable virus genomes including the additional introduced mCLDN6-gene cassette as confirmed via sequencing (data not shown). Moreover, expression of mCLDN6 demonstrated by Western blot analysis of P10 virus was comparable to the expression in cells infected by P3 vaccines (Fig. 4a), and CLDN6-VLPs revealed considerable and specific staining for CLDN6, thereby demonstrating efficient incorporation and presentation of mCLDN6 by MLV-VLPs. In contrast, no staining was found on naked VLPs expressed by cells infected with MV_vac2_-gag, that did not express the antigen demonstrating specificity of staining (Fig. 4b). These data were solidified by immunoblot analysis of VLP-containing supernatant fractions after density step-gradient centrifugation, revealing co-purification of considerable amounts of CLDN6 in this fraction (data not shown).

To assess if the murine *cldn6*-gene or its expression has any impact on respective MV’s replication, virus growth was analyzed over 6 days following infection at low MOI = 0.03. For that purpose, both MVs encoding mCLDN6, i.e. MV_vac2_-CLDN6 and MV_vac2_-gag-CLDN6, were analyzed in parallel to GFP-encoding MV_vac2_-GFP control virus for their cell-associated virus titers (Fig. 4c) as well as for infectious virus released into the supernatant (Fig. 4d). Virus growth of MV_vac2_-CLDN6 was comparable to that of MV_vac2_-GFP, whereas MV_vac2_-gag-CLDN6 was delayed by approx. 24 h in growth, but finally reached maximum titers comparable to that of both MV_vac2_-CLDN6 and MV_vac2_-GFP control virus.

Thus, cloning and rescue of MVs expressing CLDN6 (presented on particles or not) was successful. The recombinant MVs expressed the inserted antigen(s) without critical impact on viral replication and revealed genetic stability. Moreover, incorporation of mCLDN6 into retroviral VLPs was also demonstrated. Therefore, both recombinant MVs encoding mCLDN6 were regarded as potential candidates for oncolytic immunotherapy.

### Tumor-associated antigen-specific cellular immunity is induced by MV_vac2_-CLDN6

**Figure 5:**
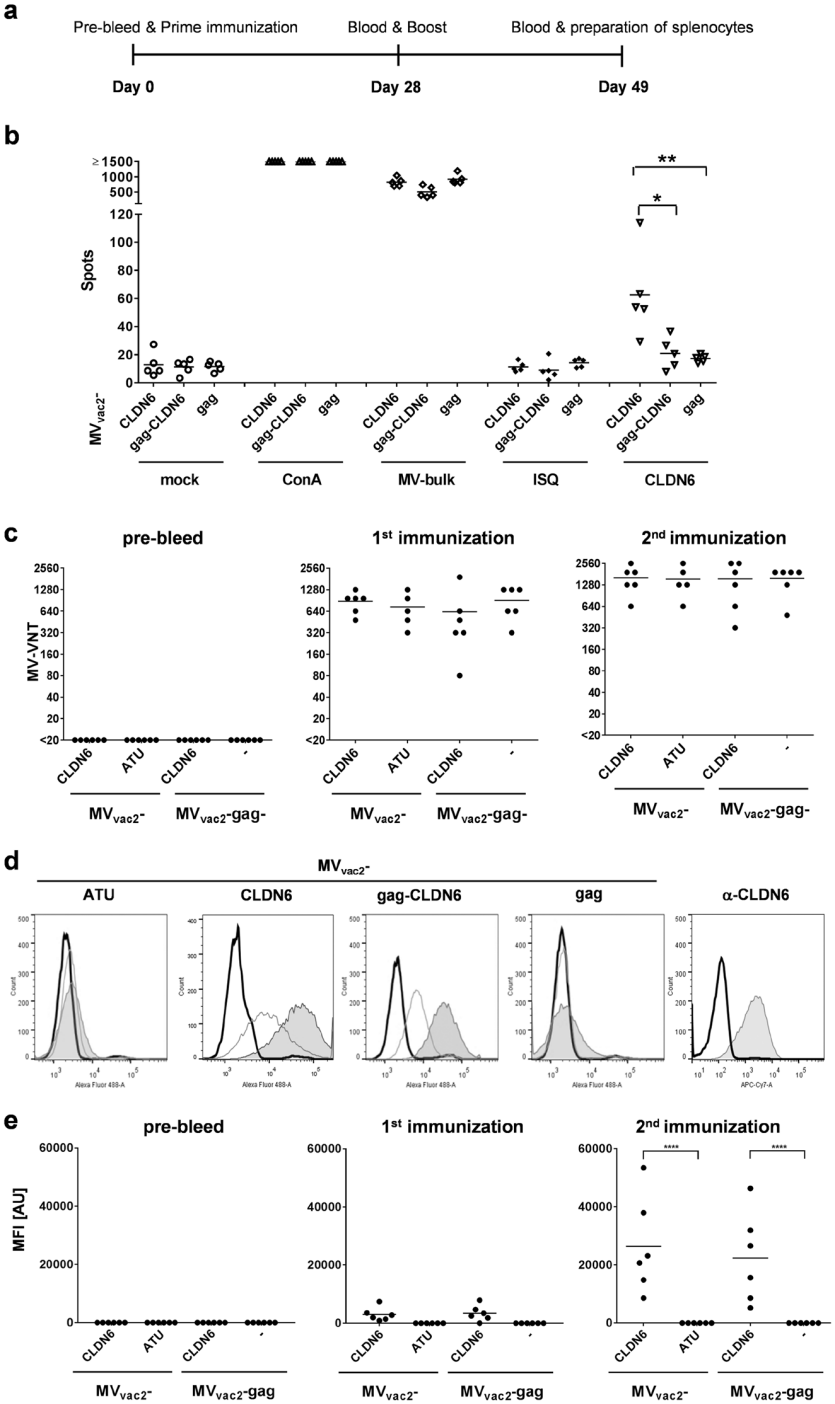
Antigen-specific cellular and humoral immunity induced by CLDN6-presenting MV. (**a**) Schematic depiction of immunization experiments. (**b**) IFN-γ ELISpot analysis using splenocytes of vaccinated mice with indicated viruses expressing CLDN6 or MLV-gag. Viruses expressing VLPs, but no antigen (MV_vac2_-gag) served as negative controls. Splenocytes isolated 21 days after boost immunization and analyzed for IFN-γ-production after incubation with ConA, viral antigen (MV bulk), specific or unspecific peptides (CLDN6 or ISQ, respectively), or without stimulation (mock). (**c**) Virus neutralizing titers (VNT) of with indicated viruses vaccinated animals’ sera were analyzed for neutralization of MV. LLOD = VNT 20. (**d**) Flow cytometry histograms of CLDN6-expressing CT26 cells stained with sera of representative mice vaccinated with indicated viruses before (solid black line), after prime (solid grey line), or after booster (filled grey area) vaccination. Staining with anti-CLDN6 mAb served as positive control (**e**) Analysis of CLDN6-specific antibody concentration in serum samples of vaccinated mice determined by FACS as shown in panel D according to MFI of stained cell populations. Dots represent single animals (n = 5–6); horizontal line represents mean per group. Statistical differences between groups were assessed by Mann-Whitney test (**b**) or two-way ANOVA (**d**); *P < 0.05; **P < 0.01; ****P < 0.0001.

To analyze the induction of CLDN6-specific cellular immune responses, splenocytes of animals vaccinated with MV_vac2_-CLDN6, MV_vac2_-gag-CLDN6, or MV_vac2_-gag control virus were analyzed for antigen-specific IFN-γ secretion by ELISpot assay. For this purpose, mice were immunized as described for Ova-encoding model vaccines (Fig. 5a) and splenocytes were analyzed by ELISpot. Splenocytes of animals vaccinated with MV_vac2_-CLDN6 revealed approx. 30 IFN-γ secreting cells per 5 × 10^5^ splenocytes after CLDN6-specific re-stimulation with overlapping CLDN6 peptides. This significant albeit moderate induction of CLDN6-specific immune cells indicated breaking of immune tolerance by recombinant MV. Splenocytes of animals vaccinated with MV_vac2_-gag-CLDN6 revealed just about 20–25 IFN-γ secreting CLDN6-specific cells per 5×10^5^ splenocytes, which is slightly above background of about 15 IFN-γ secreting cells as revealed after unspecific control stimulation and in splenocytes of mice immunized with MV_vac2_-gag. Basic T-cell reactivity was not impaired by immunization as shown by stimulation with MV bulk antigens or ConA treatment (Fig. 5b).

### Tumor-associated antigen-specific humoral immunity is induced by CLDN6-presenting MV

To assess antigen-specific humoral immune responses, sera of immunized mice were first tested for anti-vector immunity by analyzing the prevalence of anti-MV neutralizing antibodies. Mice immunized with any recombinant MV developed similar VNTs between 320 and 2,560 already after the first immunization, and were further boosted by the second immunization. These data demonstrate successful vaccination of all animals by the respective MV-derived vaccine candidates (Fig. 5c).

To determine the induction of CLDN6-specific humoral immune responses, CLDN6-binding antibodies in the sera of vaccinated mice were analyzed by flow cytometry. For this purpose, syngeneic murine CT26 cells that endogenously express CLDN6 on their surface were incubated with the individual sera of immunized mice. Bound serum antibodies were then detected by flow cytometry by using AlexaFluor488-labelled secondary antibodies directed against murine IgG for marking. Control viruses MV_vac2_-ATU and MV_vac2_-gag did not induce any antibodies that bound to CT26 cells, demonstrating specificity of the assay (Fig. 5d,e). In contrast, mice immunized with MV presenting mCLDN6 on the surface of *in situ* produced VLPs (MV_vac2_-gag-CLDN6) or MV_vac2_-CLDN6 revealed significant induction of CLDN6-specific antibodies in the immunized mice using the prime-boost protocol, with considerable boosting of antibody responses by the second immunization (Fig. 5d,e).

Given the high sequence homology of CLDN6 and CLDN9, the humoral immune reactivity of immunized mice against CLDN9-expressing cells was assessed to check for cross-reactivity. Such reactivity may be a therapeutic issue by causing off-target side-effects of the therapy on CLDN9-expressing tissue such as the inner ear^37^. Therefore, flow cytometric analysis analogous to that applied for detection of CLDN6-binding antibodies was performed. Sera of mice immunized with MV_vac2_-CLDN6, MV_vac2_-ATU, MV_vac2_-gag-CLDN6, or MV_vac2_-gag were used to stain CHO-CLDN9 cells stably expressing human CLDN9, which is 97% homologous to murine CLDN9, 70% to murine CLDN6, and 72% to human CLDN6. No CLDN9-specific antibodies were observed in sera in any experimental group, except a weak reactivity of one mouse immunized with MV_vac2_-gag-CLDN6 (Supplementary Figure S1).

In summary, the MV-derived tumor-vaccines were able to induce immunity against the endogenous TAA CLDN6. Here, moderate CLDN6-specific cellular responses were predominantly observed in mice immunized with MVs that present CLDN6 as an integral membrane protein on infected cells.

CLDN6-specific humoral immune responses were induced after vaccination with MVs that present CLDN6 on the surface of *in situ* produced VLPs as well as with MV expressing only membrane-bound CLDN6.

### Auto-antibodies induced by immunization with CLDN6 target positive cells by CDC

**Figure 6:**
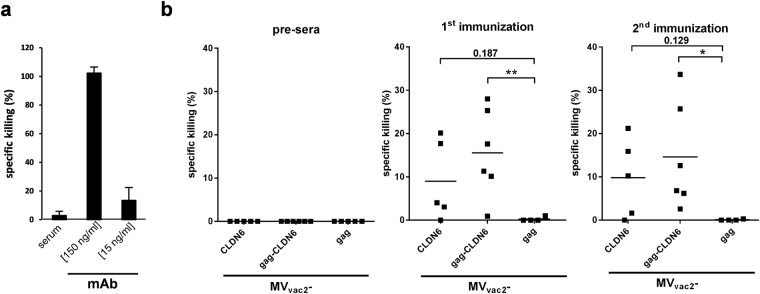
Effector functions of humoral immunity. (**a**) As positive control for CDC, a CLDN6-specific mAb was used in indicated concentrations in parallel to irrelevant serum without CLDN6-specific antibodies for testing killing by CDC assay using XTT substrate. CHO-K1-cells stably transfected with the human CLDN6 isoform served as target cells and human serum as a source for complement factors to analyze CDC-mediated lytic activity. The percentage of cell lysis is indicated with reference to completely lysed cells. (**b**) Auto-antibody mediated CLDN6-specific cell killing. CHO-K1-cells stably transfected with the human CLDN6 isoform were incubated with sera from immunized mice and tested for CDC. Statistical differences between groups were assessed by two-way ANOVA and Tukey’s test. *P < 0.05; **P < 0.01.

Vaccination-induced serum anti-CLDN6 antibodies were analyzed upon their capability to force target cell-specific lysis through Fc-mediated immune effector mechanisms, such as complement-dependent cytotoxicity (CDC). XTT-based *in vitro* assays using hCLDN6-transfected cells have been performed and the target-specific killing of cells was analyzed compared to a recombinant CLDN6-specific monoclonal antibody (positive control), the latter revealing the remarkable potential of CLDN6-specific antibodies to provoke Fc-mediated cytotoxicity (Fig. 6a). Indeed, sera of all mice vaccinated with MV_vac2_-gag-CLDN6 showed target-specific CDC with lytic activities (Fig. 6b). Also sera of MV_vac2_-CLDN6-vaccinated mice induced Fc-mediated lysis of hCLDN6-transfected cells, but revealed somewhat weaker lysis as well as one non-responder among 5 animals.

Thus, antibodies induced by MV-derived vaccines against encoded TAA elicit auto-antibodies that kill TAA-expressing cells by CDC, thereby targeting antigen-positive tumor cells as a potential effector mechanism. Interestingly, this antibody function became only significant for the sera induced by immunization with MV encoding particle-associated TAA,

### Anti-CLDN6 immunity induced by MV vectors significantly inhibits formation of lung metastases after challenge with syngeneic tumor cells

Potency of the CLDN6-specific antibody responses induced by immunization with recombinant MV_vac2_-CLDN6 or MV_vac2_-gag-CLDN6 was further investigated in a highly aggressive lung metastasis model. For this purpose, B16-F10-derived cell clones stably expressing mCLDN6 and the MV vaccine strain receptor hCD46 (to be susceptible both for MV oncolysis and immunotherapy targeting CLDN6) were generated by transduction with lentiviral vectors encoding the respective transgenes. Expression of proteins was revealed via flow cytometry using mCLDN6- as well as hCD46-specific antibodies (Supplementary Figure S2a). A single cell clone that expressed both proteins in high amounts was inoculated s.c. into the flanks of syngeneic IFNAR^−/−^-CD46Ge mice. Effective tumor growth was monitored, the tumors were resected, and stable expression of mCLDN6 and hCD46 after *in vivo* passage was demonstrated (Supplementary Figure S2b).

**Figure 7:**
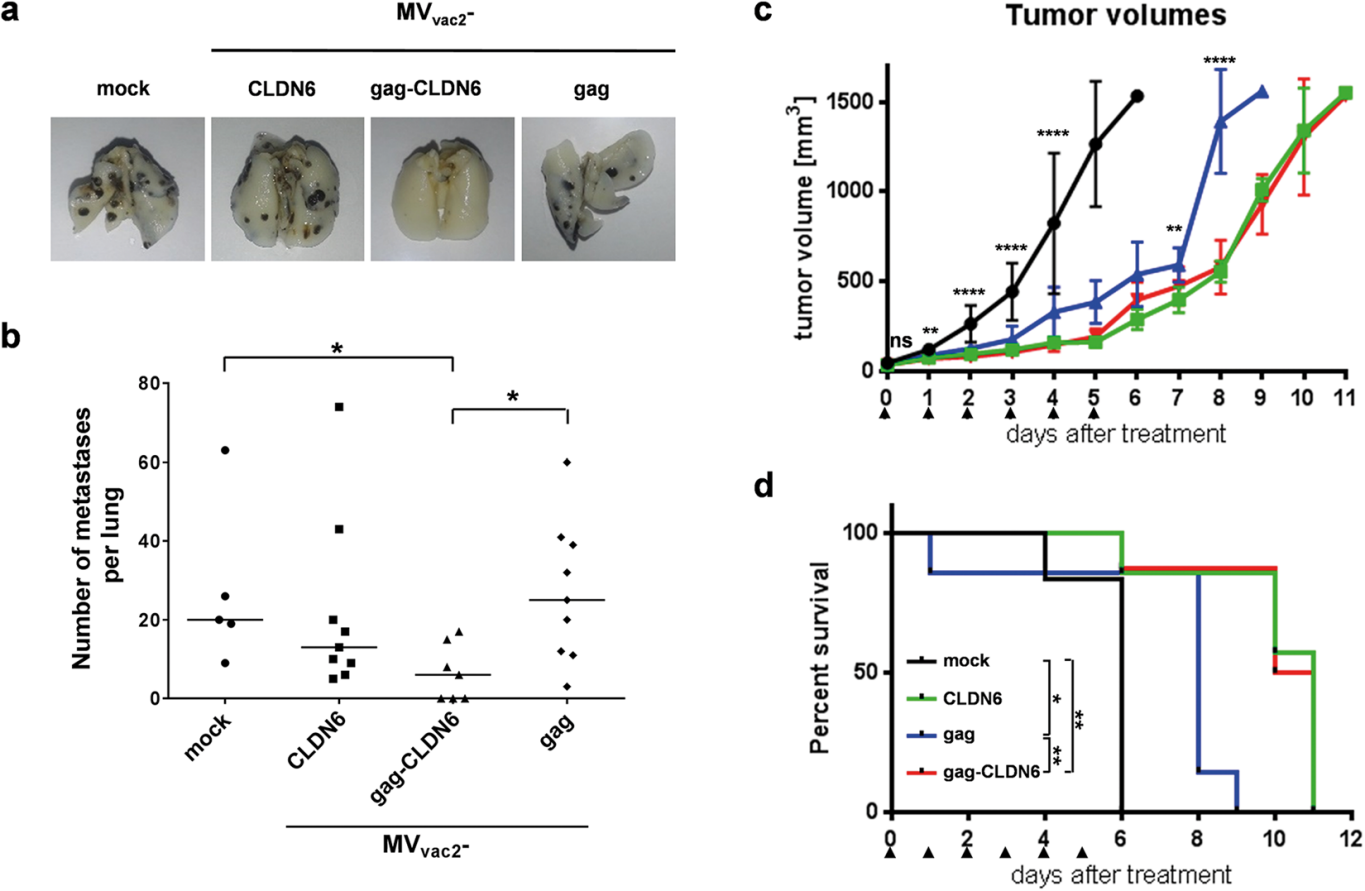
Anti-tumoral efficacy of recombinant viruses. Anti-tumoral efficacy after (**a,b**) prophylactic vaccination against metastatic spread or (**c**,**d**) therapeutic treatment of pre-established tumors. (**a**) Lungs of representative mice vaccinated twice with indicated viruses or medium (mock), 19 days after tumor-challenge. Lungs were prepared, fixed, and bleached with Fekete’s solution. B16 cell metastases become visible as black dots. (**b**) Quantitative analysis of metastases per lung after vaccination and B16-challenge. Dots represent number of metastases in individual animals (n = 5–9); horizontal line represents median per group. Statistical differences between groups were assessed by Mann Whitney test. *, P < 0.05. (**c**) Growth of subcutaneous B16-mCLDN6-hCD46 tumors on the flanks of IFNAR^−/−^-CD46Ge mice after treatment (arrow heads) with MV_vac2_-gag (blue), MV_vac2_-CLDN6 (green), MV_vac2_-gag-CLDN6 (red), or OptiMEM as mock control group (black). Mean tumor size and SD are indicated. Statistical differences between groups were assessed by one-way ANOVA. ns, non-significant; **, P < 0.01; ****, P < 0.0001. (**d**) Survival of tumor-bearing animals. Kaplan-Meyer survival plots of animals depicted in (**c**). Statistical differences between groups were assessed by Log-rank (Mantel-Cox) test. *P < 0.05; **P < 0.01.

For protection studies, IFNAR^−/−^-CD46Ge mice were immunized twice with MV_vac2_-CLDN6, MV_vac2_-gag-CLDN6, MV_vac2_-gag (vector control), or medium, alone (mock). 21 days after the second immunization, the mice were challenged i.v. with the B16-mCLDN6/hCD46 cell clone, which forms lung metastases (Fig. 7a), that were quantified *ex vivo* 19 days after challenge (Fig. 7b). Macroscopic analysis of the lungs of mice vaccinated with MV_vac2_-gag-CLDN6 revealed a significantly reduced number of metastases as compared to MV_vac2_-gag immunized or mock-treated mice (Fig. 7a,b). Of note, 3 out of 7 mice of the MV_vac2_-gag-CLDN6 group were free of any metastatic nodules in the lung, while in the other groups robust tumor growth was evident in all animals (in total n = 23). MV_vac2_-CLDN6 immunized mice revealed somewhat reduced counts of metastatic nodules in their lungs, which was however not statistically significant against mock or virus controls (Fig. 7b).

In conclusion, these data show that prophylactic vaccination with MV_vac2_-gag-CLDN6 strongly inhibits and can even protect mice against highly aggressive B16-mCLDN6/hCD46 cells forming lung metastasis after i.v. application of tumor cells mimicking pulmonary spread via the hematogenous route.

### Encoding of additional tumor antigens enhances the therapeutic efficacy of oncolytic MV

Finally, therapeutic efficacy of oncolytic MVs additionally encoding CLDN6 as TAA (MV_vac2_-CLDN6 and MV_vac2_-gag-CLDN6) was assessed in direct comparison to the efficacy of MV_vac2_ including the potential unspecific immunostimulatory effects resulting from the release of VLPs (MV_vac2_-gag) in a largely immunocompetent model with pre-established tumors. For this purpose, B16-mCLDN6/hCD46 cells of the selected clone were inoculated s.c. into the flanks of IFNAR^−/−^-CD46Ge mice. When the tumors reached a size of approx. 50 mm^3^, treatment was started by injection of each 1 × 10^6^ TCID_50_ of MV_vac2_-CLDN6, MV_vac2_-gag-CLDN6, or MV_vac2_-gag control virus on 5 consecutive days. A fourth cohort was treated by injection of similar volumes of medium without a therapeutic substance as treatment control (mock).

Tumors in the control group grew aggressively, and all mice had to be sacrificed by day 6 after initiation of treatment (Fig. 7c,d). Growth of tumors was significantly inhibited in the cohort that was treated with MV_vac2_-gag (Fig. 7c), resulting in a significantly longer survival of these mice (Fig. 7d). Cohorts treated with MV_vac2_-CLDN6 or MV_vac2_-gag-CLDN6 experienced an even stronger delay in tumor growth (Fig. 7c) and a further increase of median survival (11 vs. 9 days post initiation of treatment). These enhancements of therapeutic efficacy of both antigen-encoding MV were statistically significant in comparison to MV_vac2_-gag not encoding the specific tumor antigen, even if not taking into account the mouse of the MV_vac2_-gag cohort that prematurely died on day 2, revealing the beneficial effect of additionally equipping oncolytic MV with an anti-tumoral vaccine component.

## Discussion

The aim of cancer immunotherapy is the induction of a powerful and persisting anti-tumor immune stimulation. Viruses, also being developed as cancer treatment options (so called oncolytic viruses) are in general very immunogenic. Here, oncolytic MVs have been generated that encode the model antigen Ova or CLDN6 as an ideal TAA to be expressed either membrane-bound, only, or additionally presented on the surface of *in situ* produced VLPs. The recombinant viruses stably expressed the desired antigens and VLPs, and cellular and considerable humoral anti-TAA immune responses were induced. Here, use of the model antigen Ova allowed determination of CD8^+^-responses as the major T-cell responses induced in our system, but also discrimination between CD4^+^ T-cell responses induced by cell- or VLP-presented antigen. The TAA-encoding MVs were significantly more efficacious in treating pre-established highly aggressive tumors than control oncolytic MV and furthermore also significantly inhibited metastasis formation in prophylactic mouse tumor models. These results confirm the initial hypothesis that recombinant measles virus designed for the use in virotherapy can be further equipped for immunotherapeutic purposes, thereby significantly enhancing the immunotherapeutic mode of action of MV oncolytic therapy.

To analyze such immunotherapeutic effects, an at least partially immunocompetent animal model is necessary. For MV-driven virotherapy, this has been largely hampered by the resistance of mice and most murine cells against MV infection already at the level of cell entry, since murine MV receptor homologues are not^38,39^ or less well^40^ recognized by the virus. Therefore, MV-based virotherapy has largely been analyzed in immunodeficient mice bearing human xenograft tumors. To analyze oncolytic MV in an immunocompetent mouse tumor model, entry restriction has been partially overcome by using re-targeted MV and target-transgenic murine tumor cell lines^10,12^. This system however not allowed the direct natural interaction^41^ of replicating MV with immune cells due to the lack of respective MV receptors on murine immune cells. In this study, we have developed for the first time a largely immunocompetent albeit somewhat impaired, MV-susceptible tumor-bearing mouse model that allows assessment of direct and indirect interaction of the replicating MV vector with most components of the immune system *in vivo*. This model may therefore be more predictive for immune-mediated effects although lacking the type-I IFN-driven immune-stimulatory program, which comprises a powerful stimulus for adaptive immunity during virus infection^42^. In human patients, these limitations are not as severe since MV is a primate virus and man is the natural host. Therefore, virus-host adaptation is granted, and tumor cells are known to often up-regulate the vaccine-strain receptor CD46^43^ to protect themselves from complement activity, since CD46 is the major complement regulating protein (MCP). Nevertheless, individual patients and their tumors will have to be stratified before treatment, since at least the oncolytic vaccine has to match the tumors’ antigen profile, and susceptibility of primary tumor cells to measles virus infection is most likely quite advantageous to boost the lytic mode of action of such oncolytic vaccines. By additionally encoding a TAA to be presented either on the surface of infected cells or *in situ* produced VLPs, both cellular and strong humoral anti-TAA immune responses were induced using the MV-derived platform. Of note, humoral immunity became significant every time when the VLP-presenting vaccine has been used, an observation that may be defined more in detail by future experiments. This is even most remarkable, since the immune responses have been induced in a background lacking proper stimulation of adaptive responses by the type-I IFN pathway, which is likely to be also important for gaining full anti-tumor potency upon cancer vaccination^44^, especially since IgG-types with CDC effector function, in mice pre-dominantly IgG2a or IgG2b, became induced that are known to be typically associated with Th1-type responses. Therefore, our model may underestimate the potency of the vaccine component. Nevertheless, these tumor-specific responses substantially enhanced treatment efficacy against pre-established highly aggressive tumors and significantly inhibited metastasis formation.

Importantly, the tumor vaccine properties were independent from heterologous prime-boost protocols against the tumor antigen as it has been demonstrated to be necessary for VSV- or Maraba virus-encoded tumor antigens^13,14^, where anti-TAA immunity is most likely limited by the immuno-dominance of viral antigens. As an alternative to heterologous prime-boost, co-transfer of tumor antigen-specific T-cells^45^, or expression of cDNA libraries^46^ has revealed efficacy, but this is obviously not needed when applying the MV-based vectors, despite significant immune responses also against the MV platform. Thereby, the one-component MV-based oncolytic immunotherapeutic could be introduced much easier into clinical trial protocols.

Both MV and VSV are clinically developed for virotherapeutic purposes against different tumor entities including ovarian carcinoma, which also reveal significant up-regulation of CLDN6^23,47^, and thus constitutes an ideal tumor vaccination target, too, since few auto-immunity is to be anticipated. Indeed, passive immunotherapy with anti-CLDN6 mAb (IMAB027) is currently under clinical development for patients with recurrent advanced ovarian cancer (NCT02054351). Such antibodies are effective by inducing responses such as CDC and antibody-dependent cellular cytotoxicity (ADCC) against opsonized tumor cells^34,48^. Here, we observed efficient killing of target-positive cells by CDC using the vaccine-induced antibodies of mouse sera that were most robustly induced when using VLP-encoding vaccines. The advantages of using an oncolytic vaccine to induce humoral anti-tumoral immunity compared to the application of respective mAbs may be two-fold: Firstly, oncolytic vaccines possess an additional mode of action by directly lysing tumor cells during virus replication. Secondly, the oncolytic vaccine induces polyclonal antibodies, thereby impairing resistance development of tumor cells harboring mutations of the single epitope targeted by a given mAb.

By revealing significance compared to the respective control group in all experiments only for groups treated with MV_vac2_-gag-CLDN6, these data strengthen the initial hypothesis that VLP presentation will enhance the humoral immune responses despite prevalent immune tolerance, as had been observed before for the sole application of HBcAg-based VLPs^34^ or MLV-derived VLPs^35^. Interestingly, the experiments using the vaccines encoding the DisOva model antigen may indicate some mechanistic background: Only for the VLP-encoding vaccine, induction of CD4^+^ T helper cells became significant. This T-cell help is required for efficient induction of humoral immune responses and may thus explain the high capacity of MV_vac2_-gag-CLDN6 to induce humoral responses. Of note, the additional expression of the TAA turned out to be sufficient to induce potent anti-tumoral immune responses, different to previously studied alternative oncolytic vaccine platforms, which needed an heterologous prime for induction of anti-tumoral responses^13,14^. Interestingly, antibody effector function as well as protection in the prophylactic setting became only significant using VLP-presented antigen, a quality that might be related to inducing a Th1-bias facilitating antibody class switch. Anyway, our data provide evidence that such targeted immune induction is more effective than virus replication alone, already acting by releasing stimulatory danger- and pathogen-associated motif patterns (DAMPs and PAMPs, respectively) during tumor cell lysis^49,50^. Moreover, oncolytic viruses are known to additionally stimulate anti-tumoral immunity by direct or indirect interaction via infected tumor cells with immune cells^51^. This holds especially true for MV, which naturally is a lymphotropic virus, and also vaccine strain viruses reveal a strong preference for dendritic cells and macrophages *in vivo* ^52,53^. Moreover, the stimulatory action of such infection has already been shown^49^. These properties of the viruses allow the tumor vaccine activity also in the prophylactic setting in mice without tumors.

The positive impact of an enhanced immunotherapeutic mode of action has also been demonstrated in pre-clinical models by testing recombinant MV that encoded GM-CSF as general immune stimulus^10^ or checkpoint inhibitors such as anti-CTLA4 or anti-PD-L1 antibodies^12^. GM-CSF-encoding MV mimics Talimogene laherparepvec (T-VEC, Imlygic^TM^), the first oncolytic virus approved for treatment of malignant melanoma based on the successful phase III OPTIM trial^8,9^. The therapeutic efficacy of this OV is closely linked to induction of anti-tumoral immune responses^54^. Currently, a combination of T-VEC with check-point inhibitors is in clinical trials (NCT01740297, NCT02626000) to explore a potential synergism of the inflammatory oncolytic virotherapy with inhibition of either CTLA-4 or the PD-1/PD-L1 axis for releasing the impairment of tumor-infiltrating T-cells^55^. Treatment of patients with anti-CTLA4 mAbs reveals both: a high anti-tumoral efficacy through a general release of T-cell blockade during immunotherapy^56,57^, but also the risk of off-target effects in up to 25% of all treated patients, developing high-grade immune-related adverse events^58^.

In summary, this study provides proof of concept for the approach of presenting tumor-associated antigens, especially on particles, by oncolytic, vaccine-strain derived MV as a tumor vaccine-platform. The MV vaccine strain used is tested pre-clinically and clinically as platform for the generation of vaccines against at least 17 different pathogens, while the oncolytic efficacy is analysed in at least 7 clinical phase I or II trials. The recombinant vaccines provide powerful and protective immune responses in appropriate animal models and clinical testing. In this study, first evidence is provided that the MV platform combining both the oncolytic and tumor vaccination approach is even strong enough to break tolerance against endogenous tumor antigens and can thus be used to generate potent tumor vaccines. The potent and highly specific anti-tumoral immune responses significantly enhanced therapeutic efficacy in a novel, meaningful *in vivo* model for solid tumor growth and metastasis formation using a clinically relevant tumor antigen. Thus, oncolytic MV presenting selected TAAs may improve virotherapy efficacy by increasing the immunotherapeutic aspect of this approach.

## Methods

### Cells

Vero (ATCC CCL-81), 293 T (ATCC CRL-3216), B16-F10 (ATCC CRL-6475), CHO-K1 (ATCC CCL-61), and CT26 (ATCC CRL-2638) cell lines were purchased from ATCC (Manassas, VA, USA) and were cultured as described before^16,59^ for no longer than 6 months after thawing of the original stock. Transgenic B16-mCLDN6/hCD46 cells were cultured in DMEM-F12 GlutaMAX with 10% FBS, hygromycin B (0.4 mg/ml; Invitrogen), and puromycin (1.5 μg/ml; Invivogen, Toulouse, France). CHO-hCLDN6 or CHO-hCLDN9 cells were cultured in DMEM-F12 GlutaMAX supplemented with 10% FBS and hygromycin B (1.5 mg/ml) or blasticidine (10 μg/ml), respectively.

### Plasmids

The murine CLDN6 (mCLDN6) gene was synthesized (Geneart, Regensburg, Germany) and inserted into pENTR1A (Invitrogen) to yield pE-mmCldn6. pE-mmCldn6 or pET-15B-Ova^60^ were used to amplify *cldn6* and *ova* open reading frames (ORFs) by PCR. *cldn6* was flanked with *Mlu*I*/Aat*II sites and sequenced after cloning into pCR2.1-TOPO that yielded pCR2.1-mCLDN6. To obtain membrane-associated Ovalbumin, *ova* was amplified by PCR without STOP, flanked with *Bgl*II*/Sac*II sites, and sequenced after cloning into pCR2.1-TOPO to yield pCR2.1-OvaBgl/Sac. The *ova* fragment was introduced via *Bgl*II*/Sac*II into pDis-mod encoding for PDGFR-TM. pDis-mod is a modification of pDisplay (Invitrogen), with *Mlu*I and *Aat*II sites 5′ of the SP and 3′ of the PDGFR-TM-sequence, respectively. mCLDN6 or DisOva were inserted into p(+)MV_vac2_-ATU(P)^61^ via *Aat*II*/Mlu*I to yield p(+)MV_vac2_-mCLDN6(P) or p(+)MV_vac2_-DisOva(P), respectively. A CMV promoter cassette^62^ was inserted via *Not*I/*Sbf*I to generate p(+)PolII-MV_vac2_-mCLDN6(P) or p(+)PolII-MV_vac2_-DisOva(P). To generate genomes of VLP-encoding MV, the murine leukemia virus (MLV) *gag* gene was amplified and equipped with flanking *Mlu*I*/Aat*II sites by PCR and cloned into p(+)MV_vac2_-GFP(NR0), derived from pB(+)MVvac2^61^ with an ATU encoding GFP in pre-N to yield p(+)MV_vac2_-MLVgag(N). p(+)PolII-MV_vac2_-MLVgag(N) was constructed by inserting the promoter cassette from p(+)PolII-MV_vac2_-MERS-S(H)^16^ into p(+)-MV_vac2_-MLVgag(N) via *Psr*I. The fragment with CMV promoter and *gag* gene cassettes were transferred via *Not*I/*Sbf*I into p(+)MV_vac2_-mCLDN6(P) or p(+)MV_vac2_-pDisOva(P) to obtain p(+)PolII-MV_vac2_-MLVgag(N)-mCLDN6(P) or p(+)PolII-MV_vac2_-MLVgag(N)-DisOva(P), respectively. Details on primers and PCR protocols are available upon request.

A lentiviral transfer vector encoding mCLDN6 controlled by hEF1α promoter was generated by shuttling *mcldn6* ORF into pLenti6.4/R4R2/V5-DEST together with the EF1α promoter cassette of pENTR5′ using Gateway® cloning (Invitrogen). Using conventional cloning techniques, the vector was further modified to encode a hygromycin-T2A-GFP fusion protein downstream of the hPGL promoter to yield pL64B42E(EF1α-mCldn6)Hygromycin-T2A-GFP. Likewise, the PCR-amplified ORF of hCD46 BC1 was cloned together with an IRES-puro^R^ cassette into pSEW^63^ to yield pSEW-hCD46-IRES-puro.

### Production of lentiviral vectors

Lentiviral vectors (LVs) were produced and titered via flow cytometry in 293 T cells using a third generation LV vector system^64^ with transfer vector plasmids pL64B42E(EF1α-mCldn6)Hygromycin-T2A-GFP or pSEW-CD46-IRES-Puro and widely established protocols as described, before^65^.

### Generation of antigen-expressing cell lines

For stable co-expression of mCLDN6 and hCD46, B16-F10 target cells were transduced using LV vector-mediated gene transfer and subsequently selected for encoded antibiotic resistance. Single cell clones were separated with cloning rings and screened via flow cytometry. CHO-hCLDN6 or CHO-hCLDN9 cells were generated by LV-mediated gene transfer into CHO-K1cells. Transduced cells were selected with hygromycin B (1.5 mg/ml; Invitrogen).

### Viruses and sequence analysis thereof

Recombinant MV were rescued and passaged up to passage 10 (P10) as described^16^ and stored at −80 °C.Virus titers were determined by TCID_50_ titration according to the method of Kaerber and Spaerman^66^. The RNA genomes of recombinant MV in P3 or P10 were analyzed as described before^16^.

### Western Blot analysis

For Western Blot analysis, Vero cells cultured in 6-wells were lysed two days post infection (MOI = 0.03) and immunoblotted as previously described^67^. A polyclonal antibody reactive against full-length CLDN6 (1:100) (IBL Co., LTD., Gunma, Japan) was used as primary antibody for mCLDN6, a rabbit anti-Ovalbumin polyclonal antibody (1:20,000) (Novus Biologicals, CO, USA), a rabbit anti-MV-N polyclonal antibody (1:25,000) (Abcam, Cambridge, UK) for MV-N, and a goat α-rauscher leukemia virus (RLV) p30 antibody (1:20,000) (ATCC). HRP-coupled donkey anti-rabbit IgG (H&L) polyclonal antibody (1:10,000) (Rockland, Gilbertsville, PA) and a HRP-coupled rabbit anti-goat polyclonal antibody (1:10,000) (Dako, Glostrup, Denmark) served as secondary antibodies, as appropriate.

### Immunoelectron microscopic analysis of virus-like particles (VLPs)

VLPs were produced by infection of Vero cells with the respective MV. After 72 h, the supernatant of infected cultures was cleared by centrifugation (1,200 rpm, 3 min, 4 °C) and filtration (0.45 μm). VLPs were pelleted (25,000 × g, 2 h, 4 °C) and fixed in 100 μl PBS, 4% paraformaldehyde. VLPs were prepared for and analysed by immunoelectron microscopy as described^35^ using anti-CLDN6 monoclonal antibody (1:300; Ganymed Pharmaceuticals AG, Mainz, Germany) or pool sera of Ova-vaccinated mice (1:2,500; Paul-Ehrlich-Institut, Langen, Germany).

### Animal experiments

All animal experiments were carried out in compliance with the regulations of the German animal protection law and have been authorized by the RP Darmstadt. Six- to 12-week-old IFNAR^−/−^-CD46Ge mice^68^ were inoculated intraperitoneally with 1 × 10^5^ TCID_50_ of virus or controls on days 0 and 28, in parallel taking serum samples. Splenocytes and sera were prepared after euthanization on day 49. For intravenous tumor-challenge, immunized mice were challenged on day 49 by injection of 2 × 10^5^ B16-mCLDN6/hCD46 cells and were euthanized 19 d after challenge. Lungs were prepared, fixed, and bleached simultaneously with Fekete’s solution^69^. For tumor treatment, mice were inoculated with 1 × 10^6^ B16-mCLDN6-hCD46 cells into the right flank and treated as described, before^59^. Tumor growth was closely monitored and mice were sacrificed when the tumors reached a volume of 1,500 mm^3^.

### Antibody ELISA

Ova-specific IgG antibody titers were determined as described^70^ with the following modifications: Unspecific binding was blocked using casein-based blocking buffer (Sigma-Aldrich), and sera were tested pre-diluted 1:100 in blocking buffer. HRP-conjugated secondary goat anti-mouse IgG antibody (1:15,000; Jackson ImmunoResearch, Newmarket, UK) was used for detection. For non-linear regression analysis (curve fit), a standard curve was generated using anti-Ovalbumin mAb A6075 (Sigma-Aldrich) in serial dilutions. The IgG concentration of samples was calculated from ΔOD values using GraphPad Prism.

### FACS binding assay

Antibody binding to native epitopes was analyzed by FACS as described^34^ with CT26 (mCLDN6) or CHO-hCLDN9 cells as targets. Non-purified serum samples and an AlexaFluor488-conjugated goat-anti-mouse IgG secondary antibody (Jackson ImmunoResearch) were used for staining, while using FACSCantoII equipment (BD Biosciences, Heidelberg, Germany).

### Neutralization Assays

Virus neutralizing titers (VNT) were determined as described^16^.

### ELISpot Assays

Murine IFN-γ ELISpot assays were performed as described^60^ using 5 μg/ml per peptide of an overlapping mCLDN6 peptide mix (JPT Peptide Technologies GmbH, Berlin, Germany) for re-stimulating CLDN6-specific T-cells. The specificity of mCLDN6 reactivity was controlled by using 5 μg/ml ISQ-peptide for stimulation. Wells with too many spots to be separated were set to 750 spots (maximum spot count reliably determined).

### CDC-assay

CHO-hCLDN6 cells plated in 96-well plates were incubated with 1:10 diluted sera from immunized animals and active human serum, as a complement source, in triplicates for 80 min at 37 °C. In control wells, maximum lysis was achieved with 8% (v/v) Triton X-100 in PBS. Lytic activity was measured using XTT substrate according to the manufacturer’s instruction (Cell proliferation kit II (XTT); Roche, Mannheim, Germany) and was calculated as follows: Specific lysis [%] = [1 − (L_exp_ − L_max_)/(L_min_ − L_max_)] × 100, where L_exp_ is the absorbance of each sample at 450/620 nm, L_max_ is the absorbance of the control and L_min_ is the absorbance of the medium control.

### Immunohistochemistry (IHC)

IHC analysis was described previously^71^ on 3 μm tissue sections of xenograft tumors using primary rabbit anti-CLDN6 antiserum (0.3 μg/ml; IBL, Hamburg, DE) or anti-CD46 antibody ab108307 (11 ng/ml; Abcam, Cambridge, UK) followed by incubation with PowerVision polymer HRP-conjugated anti-rabbit secondary antibody (Immunologic, Duiven, NL).

### Statistical analysis

Statistical analysis was performed with GraphPad Prism software (GraphPad Software, Inc., La Jolla, CA). The significance between different groups was assessed using One-way ANOVA, non-parametric Mann-Whitney U test, and two-way ANOVA with Tukey post-test as specified in the respective figure legends.

### Data availability

The datasets generated during and/or analysed during the current study are available from the corresponding author on reasonable request.

## Electronic supplementary material

Supplementary Figures

## References

[1] Kreiter S (2015). Mutant MHC class II epitopes drive therapeutic immune responses to cancer. Nature.

[2] The FUTURE II Study Group. Quadrivalent vaccine against human papillomavirus to prevent high-grade cervical lesions. *The New England journal of medicine* **356**, 1915–1927 (2007). 10.1056/NEJMoa061741 17494925

[3] Palucka K, Banchereau J (2013). Dendritic-cell-based therapeutic cancer vaccines. Immunity.

[4] Kantoff PW (2010). Sipuleucel-T immunotherapy for castration-resistant prostate cancer. The New England journal of medicine.

[5] Sahin U, Kariko K, Tureci O (2014). mRNA-based therapeutics–developing a new class of drugs. Nature reviews. Drug discovery.

[6] Miest TS, Cattaneo R (2014). New viruses for cancer therapy: meeting clinical needs. Nature reviews. Microbiology.

[7] Russell SJ, Peng K-W, Bell JC (2012). Oncolytic virotherapy. Nature biotechnology.

[8] Greig SL (2016). Talimogene Laherparepvec: First Global Approval. Drugs.

[9] Andtbacka RHI (2015). Talimogene Laherparepvec Improves Durable Response Rate in Patients With Advanced Melanoma. Journal of clinical oncology: official journal of the American Society of Clinical Oncology.

[10] Grossardt C (2013). Granulocyte-macrophage colony-stimulating factor-armed oncolytic measles virus is an effective therapeutic cancer vaccine. Human gene therapy.

[11] Grote D, Cattaneo R, Fielding AK (2003). Neutrophils contribute to the measles virus-induced antitumor effect: enhancement by granulocyte macrophage colony-stimulating factor expression. Cancer research.

[12] Engeland CE (2014). CTLA-4 and PD-L1 checkpoint blockade enhances oncolytic measles virus therapy. Molecular therapy: the journal of the American Society of Gene Therapy.

[13] Bridle BW (2009). Vesicular stomatitis virus as a novel cancer vaccine vector to prime antitumor immunity amenable to rapid boosting with adenovirus. Molecular therapy: the journal of the American Society of Gene Therapy.

[14] Pol JG (2014). Maraba virus as a potent oncolytic vaccine vector. Molecular therapy: the journal of the American Society of Gene Therapy.

[15] Despres P (2005). Live measles vaccine expressing the secreted form of the West Nile virus envelope glycoprotein protects against West Nile virus encephalitis. The Journal of infectious diseases.

[16] Malczyk AH (2015). A Highly Immunogenic and Protective Middle East Respiratory Syndrome Coronavirus Vaccine Based on a Recombinant Measles Virus Vaccine Platform. Journal of virology.

[17] Ramsauer K (2015). Immunogenicity, safety, and tolerability of a recombinant measles-virus-based chikungunya vaccine: a randomised, double-blind, placebo-controlled, active-comparator, first-in-man trial. The Lancet. Infectious diseases.

[18] Turksen K, Troy TC (2001). Claudin-6: a novel tight junction molecule is developmentally regulated in mouse embryonic epithelium. Developmental dynamics: an official publication of the American Association of Anatomists.

[19] Abuazza G (2006). Claudins 6, 9, and 13 are developmentally expressed renal tight junction proteins. American journal of physiology. Renal physiology.

[20] D’Souza T, Sherman-Baust CA, Poosala S, Mullin JM, Morin P (2009). J. Age-related changes of claudin expression in mouse liver, kidney, and pancreas. The journals of gerontology. Series A, Biological sciences and medical sciences.

[21] Micke P (2014). Aberrantly activated claudin 6 and 18.2 as potential therapy targets in non-small-cell lung cancer. International journal of cancer.

[22] Rendón-Huerta E (2010). Distribution and expression pattern of claudins 6, 7, and 9 in diffuse- and intestinal-type gastric adenocarcinomas. Journal of gastrointestinal cancer.

[23] Stadler CR (2016). Characterization of the first-in-class T-cell-engaging bispecific single-chain antibody for targeted immunotherapy of solid tumors expressing the oncofetal protein claudin 6. Oncoimmunology.

[24] Ushiku T, Shinozaki-Ushiku A, Maeda D, Morita S, Fukayama M (2012). Distinct expression pattern of claudin-6, a primitive phenotypic tight junction molecule, in germ cell tumours and visceral carcinomas. Histopathology.

[25] Ben-David U, Nudel N, Benvenisty N (2013). Immunologic and chemical targeting of the tight-junction protein Claudin-6 eliminates tumorigenic human pluripotent stem cells. Nature communications.

[26] Sahin U (2015). First-in-human phase I/II dose-escalation study of IMAB027 in patients with recurrent advanced ovarian cancer (OVAR). Preliminary data of phase I part. Journal of Clinical Oncology.

[27] Russell SJ (2014). Remission of disseminated cancer after systemic oncolytic virotherapy. Mayo Clinic proceedings.

[28] Griffin, D. E. In *Wiley Encyclopedia of Molecular Medicine* (John Wiley & Sons, Inc, Hoboken, NJ, USA, 2002).

[29] Hilleman MR (2001). Current overview of the pathogenesis and prophylaxis of measles with focus on practical implications. Vaccine.

[30] Mossong J, O’Callaghan CJ, Ratnam S (2000). Modelling antibody response to measles vaccine and subsequent waning of immunity in a low exposure population. Vaccine.

[31] Rager-Zisman B (2003). The effect of measles-mumps-rubella (MMR) immunization on the immune responses of previously immunized primary school children. Vaccine.

[32] Wong-Chew RM, Beeler JA, Audet S, Santos JI (2003). Cellular and humoral immune responses to measles in immune adults re-immunized with measles vaccine. Journal of medical virology.

[33] Mühlebach, M. D. Vaccine platform recombinant measles virus. *Virus genes* **53**, 733–740 (2017). 10.1007/s11262-017-1486-3 PMC7089060 28710608

[34] Klamp T (2011). Highly specific auto-antibodies against claudin-18 isoform 2 induced by a chimeric HBcAg virus-like particle vaccine kill tumor cells and inhibit the growth of lung metastases. Cancer research.

[35] Bach, P. *et al*. Vaccination with Abeta-displaying virus-like particles reduces soluble and insoluble cerebral Abeta and lowers plaque burden in APP transgenic mice. *Journal of immunology (Baltimore*, *Md*.*: 1950)* **182**, 7613–7624 (2009). 10.4049/jimmunol.0803366 19494285

[36] Meyers DE, Wang AA, Thirukkumaran CM, Morris DG (2017). Current Immunotherapeutic Strategies to Enhance Oncolytic Virotherapy. Front. Oncol..

[37] Nakano Y (2009). A claudin-9-based ion permeability barrier is essential for hearing. PLoS genetics.

[38] Ono N, Tatsuo H, Tanaka K, Minagawa H, Yanagi Y (2001). V domain of human SLAM (CDw150) is essential for its function as a measles virus receptor. Journal of virology.

[39] Tsujimura A (1998). Molecular cloning of a murine homologue of membrane cofactor protein (CD46): preferential expression in testicular germ cells. The Biochemical journal.

[40] Muhlebach MD (2011). Adherens junction protein nectin-4 is the epithelial receptor for measles virus. Nature.

[41] Yanagi Y, Takeda M, Ohno S (2006). Measles virus: cellular receptors, tropism and pathogenesis. The Journal of general virology.

[42] Stetson DB, Medzhitov R (2006). Type I interferons in host defense. Immunity.

[43] Anderson BD, Nakamura T, Russell SJ, Peng K-W (2004). High CD46 receptor density determines preferential killing of tumor cells by oncolytic measles virus. Cancer research.

[44] Kranz LM (2016). Systemic RNA delivery to dendritic cells exploits antiviral defence for cancer immunotherapy. Nature.

[45] Rommelfanger DM (2012). Systemic combination virotherapy for melanoma with tumor antigen-expressing vesicular stomatitis virus and adoptive T-cell transfer. Cancer research.

[46] Kottke T (2011). Broad antigenic coverage induced by vaccination with virus-based cDNA libraries cures established tumors. Nature medicine.

[47] Wang L (2013). Clinicopathologic significance of claudin-6, occludin, and matrix metalloproteinases −2 expression in ovarian carcinoma. Diagnostic pathology.

[48] Hashimoto Y (2016). Generation and characterization of a human-mouse chimeric antibody against the extracellular domain of claudin-1 for cancer therapy using a mouse model. Biochemical and biophysical research communications.

[49] Gauvrit A (2008). Measles virus induces oncolysis of mesothelioma cells and allows dendritic cells to cross-prime tumor-specific CD8 response. Cancer research.

[50] Guillerme J-B (2013). Measles virus vaccine-infected tumor cells induce tumor antigen cross-presentation by human plasmacytoid dendritic cells. Clinical cancer research: an official journal of the American Association for Cancer Research.

[51] Guo ZS, Liu Z, Bartlett DL (2014). Oncolytic Immunotherapy: Dying the Right Way is a Key to Eliciting Potent Antitumor Immunity. Front. Oncol..

[52] Vries RDde (2010). *In vivo* tropism of attenuated and pathogenic measles virus expressing green fluorescent protein in macaques. Journal of virology.

[53] Rennick LJ (2015). Live-attenuated measles virus vaccine targets dendritic cells and macrophages in muscle of nonhuman primates. Journal of virology.

[54] Liu BL (2003). ICP34.5 deleted herpes simplex virus with enhanced oncolytic, immune stimulating, and anti-tumour properties. Gene therapy.

[55] Couzin-Frankel J (2013). Breakthrough of the year 2013. Cancer immunotherapy. Science (New York, N.Y.).

[56] Hodi FS (2010). Improved survival with ipilimumab in patients with metastatic melanoma. The New England journal of medicine.

[57] Leach DR, Krummel MF, Allison JP (1996). Enhancement of antitumor immunity by CTLA-4 blockade. Science (New York, N.Y.).

[58] Bertrand A, Kostine M, Barnetche T, Truchetet M-E, Schaeverbeke T (2015). Immune related adverse events associated with anti-CTLA-4 antibodies: systematic review and meta-analysis. BMC medicine.

[59] Friedrich K (2013). DARPin-targeting of measles virus: unique bispecificity, effective oncolysis, and enhanced safety. Molecular therapy: the journal of the American Society of Gene Therapy.

[60] Uhlig KM (2015). Lentiviral Protein Transfer Vectors Are an Efficient Vaccine Platform and Induce a Strong Antigen-Specific Cytotoxic T Cell Response. Journal of virology.

[61] del Valle JR (2007). A vectored measles virus induces hepatitis B surface antigen antibodies while protecting macaques against measles virus challenge. Journal of virology.

[62] Martin A, Staeheli P, Schneider U (2006). RNA polymerase II-controlled expression of antigenomic RNA enhances the rescue efficacies of two different members of the Mononegavirales independently of the site of viral genome replication. Journal of virology.

[63] Demaison C (2002). High-level transduction and gene expression in hematopoietic repopulating cells using a human immunodeficiency correction of imunodeficiency virus type 1-based lentiviral vector containing an internal spleen focus forming virus promoter. Human gene therapy.

[64] Zufferey R, Nagy D, Mandel RJ, Naldini L, Trono D (1997). Multiply attenuated lentiviral vector achieves efficient gene delivery *in vivo*. Nature biotechnology.

[65] Koste L (2014). T-cell receptor transfer into human T cells with ecotropic retroviral vectors. Gene therapy.

[66] Kärber G (1931). Beitrag zur kollektiven Behandlung pharmakologischer Reihenversuche. Archiv f. experiment. Pathol. u. Pharmakol.

[67] Funke S (2008). Targeted Cell Entry of Lentiviral Vectors. Mol Ther.

[68] Mrkic B (1998). Measles virus spread and pathogenesis in genetically modified mice. Journal of virology.

[69] Overwijk, W. W. & Restifo, N. P. B16 as a mouse model for human melanoma. *Current protocols in immunology* Chapter 20, Unit20.1 (2001). 10.1002/0471142735.im2001s39 PMC2763508 18432774

[70] Schulke S (2014). Prevention of intestinal allergy in mice by rflaA:Ova is associated with enforced antigen processing and TLR5-dependent IL-10 secretion by mDC. PloS one.

[71] Woll S (2014). Claudin 18.2 is a target for IMAB362 antibody in pancreatic neoplasms. International journal of cancer.

