# Supplementary material for: Antigen-specific oncolytic MV-based tumor vaccines through presentation of selected tumor-associated antigens on infected cells or virus-like particles
Source: Sci Rep. 2017 Dec 4;7:16892. doi: 10.1038/s41598-017-16928-8 (PMC5715114; doi:10.1038/s41598-017-16928-8)

Supplementary Material Hutzler et al.

Antigen-specific oncolytic MV-based tumor vaccines through presentation of selected tumor-associated antigens on infected cells or virus-like particles

Stefan Hutzler, Stephanie Erbar, Robert A. Jabulowsky, Jan R.H. Hanauer, Jürgen H. Schnotz, Tim Beissert, Bianca S. Bodmer, Regina Eberle, Klaus Boller, Thorsten Klamp, Ugur Sahin, Michael D. Mühlebach*

Supplementary Fig. S1

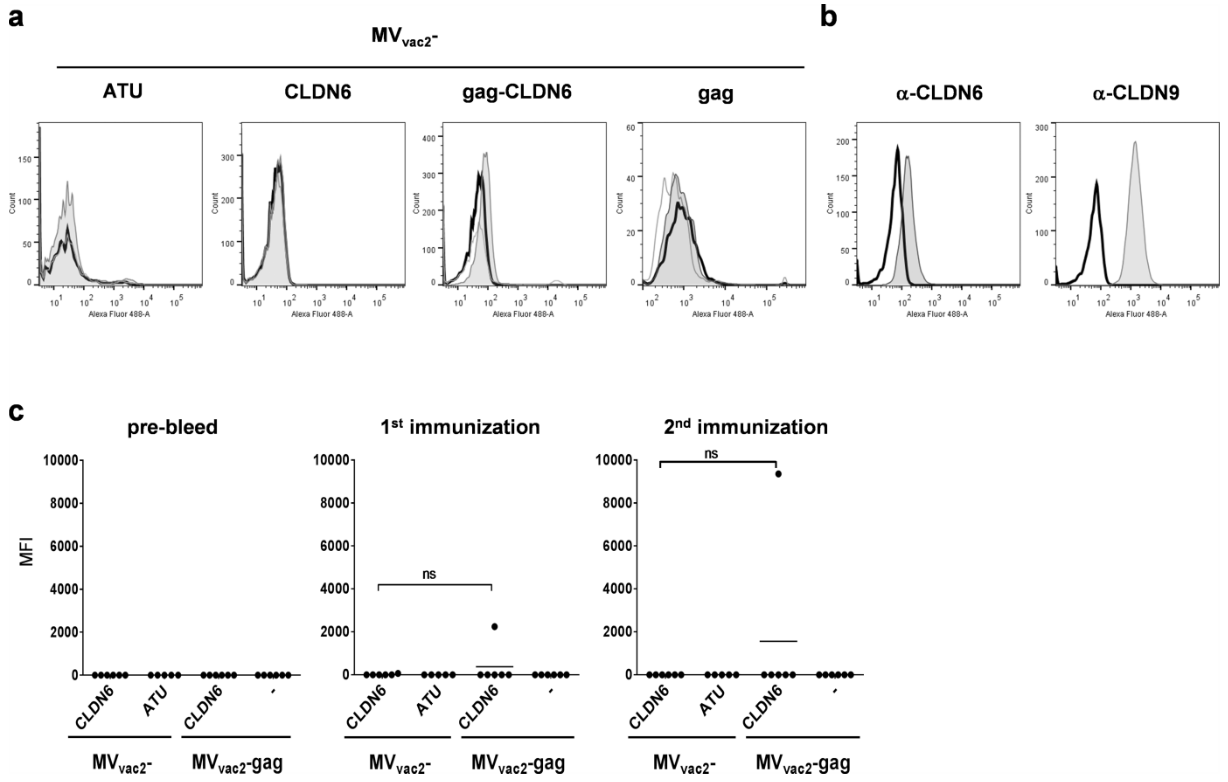

Supplementary Figure S1. Cross-reactivity of humoral immunity induced by CLDN6-expressing MV. Cross-reactivity of antibodies present in sera from immunized mice with CLDN9 were analyzed using CHO-K1 cells stably expressing human CLDN9 on the cell-surface (**a**). Flow cytometry histograms of hCLDN9-expressing CHO cells stained with sera of representative mice vaccinated with indicated viruses before (solid black line), after prime (solid grey line), or after booster (filled grey area) vaccination. (**b**) Positive control of CLDN9-expression on CHO-CLDN9 cells stained with secondary antibody only (black), CLDN6- or CLDN9-reactive hybridoma supernatant (filled grey area). (**c**) Analysis of CLDN9-specific antibody concentration in serum samples of vaccinated mice determined by FACS as shown in panel (**a**) according to MFI of stained cell populations. Dots represent single animals (n = 5-6); horizontal line represents mean per group. Statistical analysis was assessed using two-way ANOVA and Tukey´s multiple comparison test. (ns, not significant).

Supplementary Fig. S2:

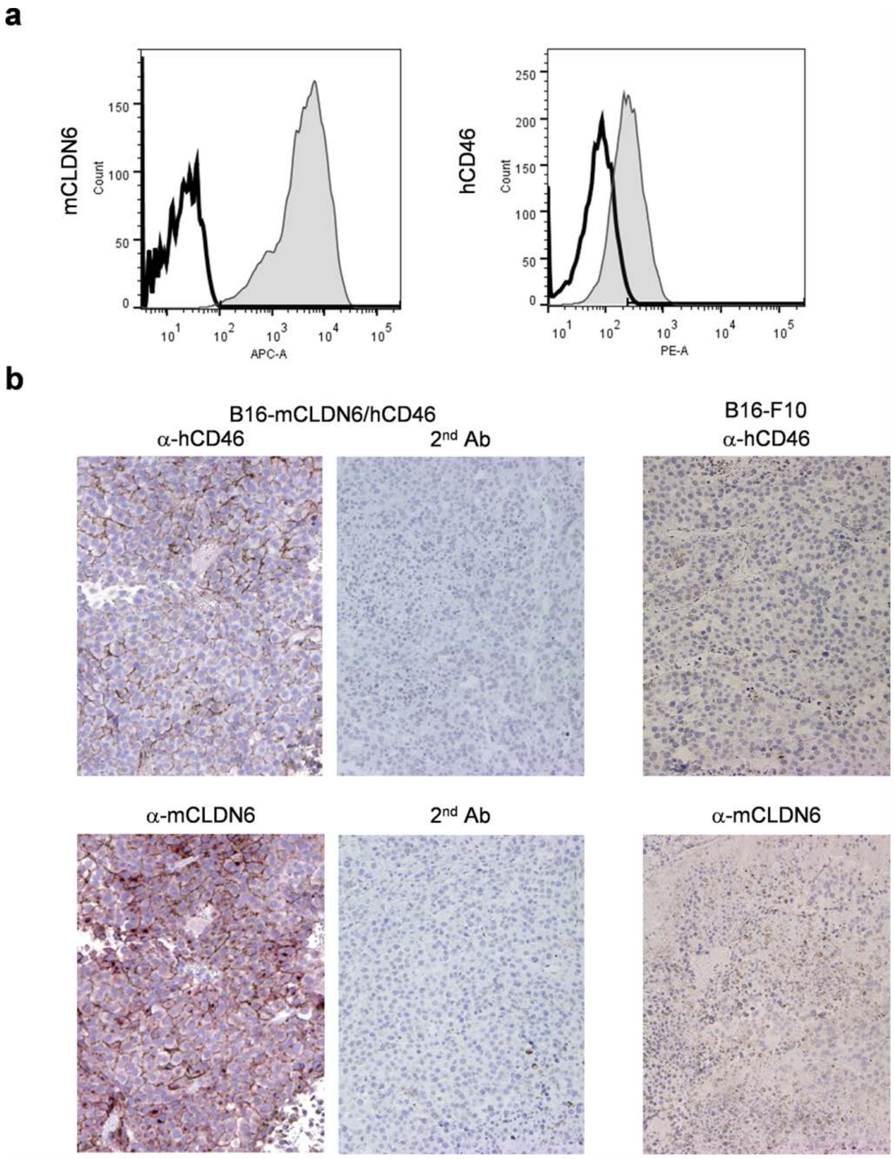

Supplementary Figure S2. Characterization of B16-mCLDN6/hCD46 cell clone. (**a**) Expression of mCLDN6 (left panel) or hCD46 (right panel) on the surface of a transgenic B16-mCLDN6-hCD46 single cell clone (filled grey area) in direct comparison to parental B16-F10 cells (black solid line) by flow cytometry. (**b**) Stability of transgene expression in B16-mCLDN6/hCD46 cell forming s.c. tumors analyzed by immunohistochemistry after tumor resection and staining for CLDN6 (upper panel) or CD46 (lower panel), in comparison to 2nd antibody control staining or B16-F10 wt tumor control stained in a separate experiment. Magnification, 200x

Supplementary Material Hutzler et al.,
Figure 2a non-cropped blots:

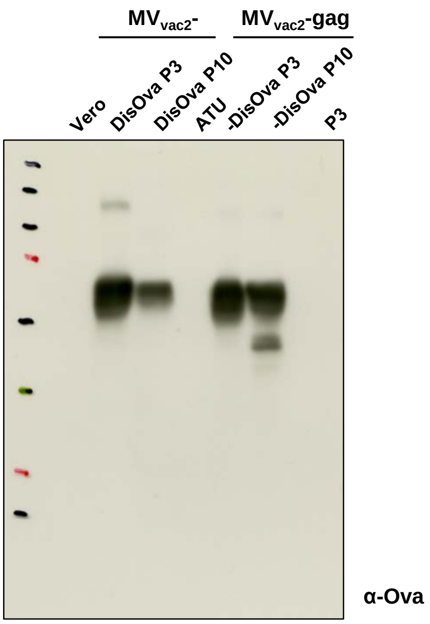

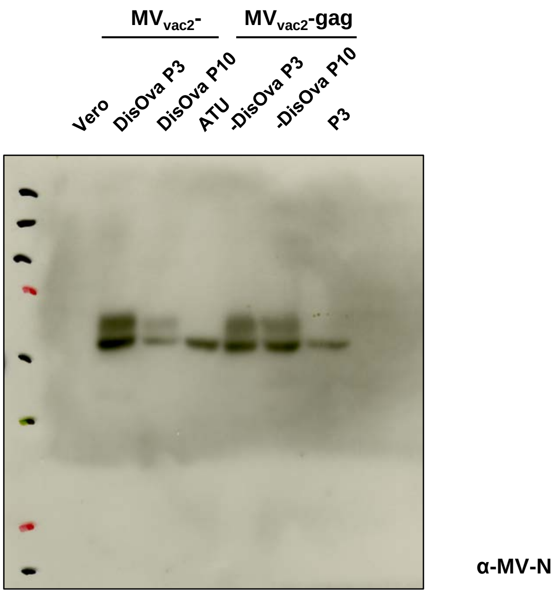

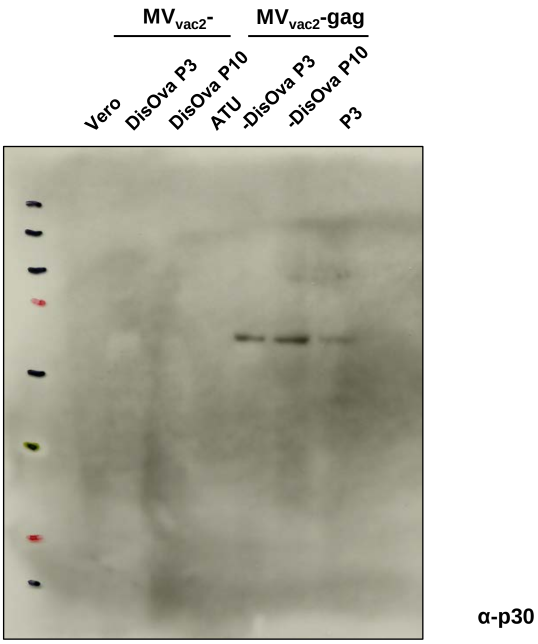

Supplementary Material Hutzler et al., Figure 4a non-cropped blots:

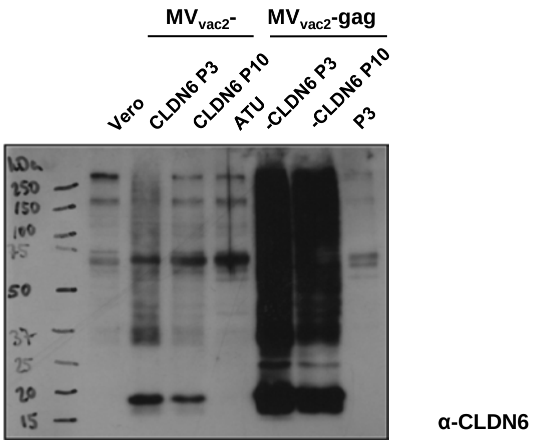

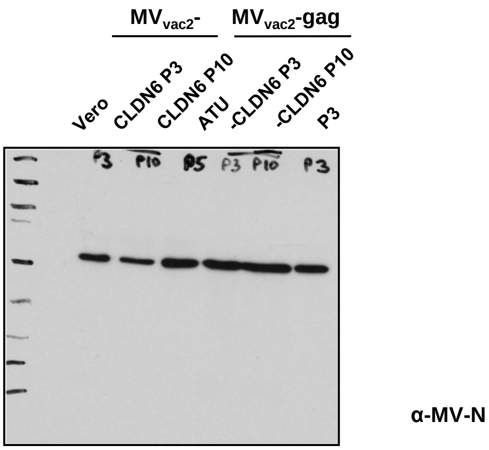

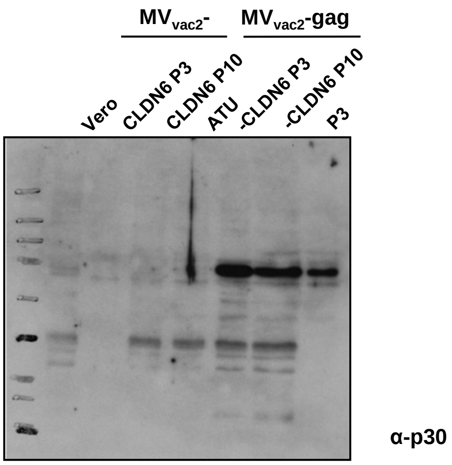

Supplement: Supplementary file 1 — Supplementary Figures [file 41598_2017_16928_MOESM1_ESM.pdf]
